# Dividing the Spoils of Growth and the Cell Cycle: The Fission Yeast as a Model for the Study of Cytokinesis

**DOI:** 10.1002/cm.20500

**Published:** 2011-01-10

**Authors:** Anupama Goyal, Masak Takaine, Viesturs Simanis, Kentaro Nakano

**Affiliations:** 1 EPFL SV ISREC UPSIMSV2.1830, Station 19, CH 1015 Lausanne, Switzerland; 2 Structural Biosciences, Graduate School of Environmental and Life Sciences, University of Tsukuba1-1-1 Tennohdai, Tsukuba, Ibaraki 305-8577, Japan

**Keywords:** cytokinesis, SIN, contractile ring, actin, myosin, *Schizosaccharomyces pombe*

## Abstract

Cytokinesis is the final stage of the cell cycle, and ensures completion of both genome segregation and organelle distribution to the daughter cells. Cytokinesis requires the cell to solve a spatial problem (to divide in the correct place, orthogonally to the plane of chromosome segregation) and a temporal problem (to coordinate cytokinesis with mitosis). Defects in the spatiotemporal control of cytokinesis may cause cell death, or increase the risk of tumor formation [Fujiwara et al., 2005 (Fujiwara T, Bandi M, Nitta M, Ivanova EV, Bronson RT, Pellman D. 2005. Cytokinesis failure generating tetraploids promotes tumorigenesis in p53-null cells. Nature 437:1043–1047); reviewed by Ganem et al., 2007 (Ganem NJ, Storchova Z, Pellman D. 2007. Tetraploidy, aneuploidy and cancer. Curr Opin Genet Dev 17:157–162.)]. Asymmetric cytokinesis, which permits the generation of two daughter cells that differ in their shape, size and properties, is important both during development, and for cellular homeostasis in multicellular organisms [reviewed by Li, 2007 (Li R. 2007. Cytokinesis in development and disease: variations on a common theme. Cell Mol Life Sci 64:3044–3058)]. The principal focus of this review will be the mechanisms of cytokinesis in the mitotic cycle of the yeast *Schizosaccharomyces pombe*. This simple model has contributed significantly to our understanding of how the cell cycle is regulated, and serves as an excellent model for studying aspects of cytokinesis. Here we will discuss the state of our knowledge of how the contractile ring is assembled and disassembled, how it contracts, and what we know of the regulatory mechanisms that control these events and assure their coordination with chromosome segregation. © 2011 Wiley-Liss, Inc.

## Introduction

We will briefly introduce the *S. pombe* model, giving references to reviews addressing aspects of fission yeast biology that are beyond the scope of this overview. As their common name implies, cells of the fission yeast *Schizosaccharomyces pombe* divide by medial fission, reminiscent of cell division of animal cells. The cells take the form of a cylinder capped by hemispherical ends. During interphase, cells grow mainly at their tips, with cell length being a measure of cell cycle progression. Upon commitment to mitosis, cells stop elongating and reorganize the actin and tubulin cytoskeletons in preparation for nuclear and cell division [McCully and Robinow,^1971^; Mitchison and Nurse,^1985^; Marks et al.,^1986^; Hagan,^1998^]. The duplicated spindle pole body (SPB) inserts into the nuclear envelope [Ding et al.,^1997^] and the interphase microtubule array is replaced with an intranuclear spindle. Three mitotic phases have been defined [Nabeshima et al.,^1998^]; phase 1, when the spindle is formed and the SPBs move to opposite sides of the nucleus; phase 2, when chromosomes are captured and aligned prior to separation, during which the spindle length does not increase much; phase 3, which corresponds to anaphase B, when the sister chromatids are separated and move to opposite SPBs, which then move apart [reviewed by Hagan,^1998^]. The position of the division site is set by the location of the interphase nucleus and depends on a PH-domain protein mid1p and proteins that confer cell polarity [reviewed by Oliferenko et al.,^2009^]. A contractile ring (CR), which is composed mainly of actin and myosin filaments, is assembled at the centre of the cell during mitosis [reviewed by Pollard,^2008^; Pollard and Wu,^2010^] at the end of anaphase, the constriction of this ring is thought to guide synthesis of the septum that bisects the cell [reviewed by Ishiguro,^1998^; Sipiczki,^2007^]. Cytokinesis is mediated by CR or actin and myosin in most eukaryotic cells, including *S. pombe*. This has led to the use of this simple model system for studies addressing the regulatory and mechanistic aspects of cytokinesis, which have provided many insights into the regulation of this critical stage of the cell cycle. Cytokinesis is regulated by a group of protein kinases known as the septation initiation network (SIN). The SIN is essential for cytokinesis, and is coregulated with mitotic events, to ensure the coordination of mitosis and cytokinesis (see below). Table I lists the major proteins of whose function is required for cytokinesis in *S. pombe*. Table II lists the principal elements and regulators of the SIN, and their functions.

**Table I:** Proteins Required for Cytokinesis in *S. pombe*

Protein	Structural features	Function
Node assembly		
mid1p/dmf1p	PH-domain protein	Shuttles between nucleus and cytoplasm and determines the division site
plo1p	Polo-like Ser/Thr protein kinase	Induces exclusion of Mid1p from nucleus and activates SIN pathway
CR assembly		
act1p	Conventional actin	Major component of CR
myo2p	Myosin II heavy chain (MHC II)	Actin-based motor activity, essential for cytokinesis
myo3p/myp2p	Unconventional MHC II	Actin-based motor activity, essential for cytokinesis under stressful condition
cdc4p	EF-hand protein	Essential light chain of myosin II, associating with rng2p and pik1p
rlc1p	EF-hand protein	Regulatory light chain of myosin II, phosphoregulated by pak1p
rng2p	IQGAP-like protein	F-actin-crosslinking, actin polymerization, controlling myo2p distribution
cdc12p	Formin	Induces actin polymerization from G-actin-cdc3p (profilin) complex
cdc15p	PCH-family, F-BAR-domain	Controls membrane domain and binds to cdc12p and myo1p
ain1p	α-actinin	Crosslinks F-actin, essential for cytokinesis under stressful condition
fim1p	Fimbrin	F-actin-bundling, unessential for CR formation
adf1p	ADF/cofilin	Accelerates actin-depolymerization and severs F-actin
cdc8p	Tropomyosin	Activates actin-myosin II interaction, protects F-actin from Adf1
rng3p	UCS-family protein	Activates motor activity of myosin II
Septum formation		
cps1p/drc1p	β-1,3-glucan synthase	Septum synthesis, a possible sensor for cytokinesis checkpoint signaling
mok1p	α-1,3-glucan synthase	Septum synthesis
eng1p	Endo-β-1,3-glucanase	Required for cell separation probably by digesting the primary septum
agn1p	Endo-α-glucanase	Required for cell separation redundantly with eng1p
mid2p	Similar to Mid1p	Localizes septin ring at a division site, required for cell separation
ace2p	Zinc finger C2H2 type domain	Induces expression of genes required for cell separation including *mid2*

**Table II:** Components and Regulators of the *S. pombe* SIN

Protein	Structural features	Function
SIN scaffold		
cdc11p	Leucine rich repeat	Scaffold subunit; interacts with sid4p,spg1p, sid2p, cdc13p and cdc16p
ppc89p	SPB protein	Interacts with sid4p
sid4p	SPB protein	Interacts with cdc11p, plo1p and dma1p
Players		
spg1p	GTPase	Interacts with cdc11p and cdc7p; spg1p-GTP binds cdc7p which process downstream signaling
cdc16p	GAP	Regulates GTP hydrolysis of spg1p
byr4p	Scaffold protein	Regulates GTP hydrolysis of spg1p
cdc7p	Ser/Thr kinase	Binds to spg1p-GTP and process downstream signaling
sid1p	PAK-related kinase	Binds to cd14p and process SIN signaling downstream to cdc7p kinase
cdc14p	SIN component	Binds to sid1p and process SIN signaling downstream to cdc7p kinase
sid2p	AGC-kinase	Binds to mob1p and is recruited to medial region at the end of anaphase thus assumed to trigger ring contraction and septum formation
mob1p	MOB1/phocein family	Binds to mob1p and is recruited to medial region at the end of anaphase thus assumed to trigger ring contraction and septum formation
Regulators		
dma1p	FHA-RING finger protein	Prevents septation in spindle assembly checkpoint-arrested cells; may regulate plo1p localization at the SPB
zfs1p	Zinc-finger RNA binding protein	Prevents septation in spindle assembly checkpoint-arrested cells; targets unknown
scw1p	RNA binding protein	Rescues sid2p mutant alleles indirectly; probably through stabilization of microtubules
par1p	Regulatory subunit of PP2A	Negative regulator of SIN pathway; targets unknown
fin1p	NIMA-related kinase	Regulates spindle formation, affinity of plo1 for the SPB and activity of SIN at old pole.
rad24p	14-3-3 protein	Retains phosphorylated flp1p in cytoplasm
sce3p	A putative RNA-binding protein that is homologous to human eIF4B	Rescues mutations in cdc11p; targets unknown
dnt1p	Nucleolar protein	Interacts negatively with the SIN proteins; function unknown
flp1p/clp1p	Phosphoprotein phosphatase	Functions in cytokinesis checkpoint CDC14 homolog
etd1p	Possible GEF	Association of cdc7p and sid2p at the SPB; degradation could be linked to inactivation of SIN

## Spatiotemporal Regulation of Cytokinesis in *S. pombe*

### Cell-Cycle Control of Cytokinesis

*S. pombe* has a single, essential mitotic cyclin-dependent kinase (CDK), named cdc2p: its function is conserved in distantly related yeasts such as *S. cerevisiae* where it is named CDC28, and in human cells, where it is known as CDK1 [Beach et al.,^1982^; Lee and Nurse,^1987^]. Cdc2p is required for mitotic entry; its activity is regulated in part by phosphorylation at Tyr15. The level of phosphorylation at this site is governed by the relative activities of the protein kinase wee1p and the phosphoprotein phosphatase cdc25p. The activities of cdc25p and wee1p are regulated at multiple levels to ensure that mitotic commitment occurs at the appropriate time; recent studies have revealed mechanisms that appear to coordinate growth, nutrient status and cell division [Petersen and Nurse,^2007^; Martin and Berthelot-Grosjean,^2009^; Moseley et al.,^2009^].

Reorganization of the actin and tubulin cytoskeletons for mitosis and cytokinesis depends upon commitment mitosis and CDK activity [reviewed by Marks et al.,^1986^; Hagan,^1998^]. Once mitosis has been initiated, elevated cdc2p activity prevents cytokinesis early in mitosis [Yamano et al.,^1996^; Chang et al.,^2001^; Dischinger et al.,^2008^]. Following CDK inactivation, cytokinesis requires the activity of a group of protein kinases known as the SIN (see below).

### Positioning of Division Plane

Unlike mammalian cells, fission yeast undergo a “closed” mitosis, in which the nuclear envelope does not break down, and the spindle forms inside the nucleus [Hagan and Hyams^1988^; reviewed by Hagan,^1998^]. A consequence of this is that the molecular mechanism controlling the positioning of the division plane differs from that in animal cells. Though microtubules are not required for cytokinesis in *S. pombe* [He et al.,^1997^,^1998^; Sparks et al.,^1999^], they do play an important role in the spatial control of the division plane. The division site corresponds to the location of the late interphase nucleus, which is kept in the middle of the cell by a microtubule-dependent mechanism [Tran et al.,^2000^]. Moreover, the localization of pom1p at the cell tips [Bähler and Pringle,^1998^] is assured by the cytoplasmic MTs in *S. pombe* [reviewed by Martin,^2009^].

The PH-domain protein mid1p, is a key factor of positioning CR at the medial cell cortex [Chang et al.,^1996^; Sohrmann et al.,^1996^; Daga and Chang,^2005^]. Mid1p shuttles between the nucleus and cell cortex [Paoletti and Chang,^2000^] and positions the division plane by acting as an “anchor” for CR proteins. At least three molecular systems control mid1p localization in a cell-cycle specific manner. The first is that cdr2p forms nodes that physically anchor a population of mid1p at cell cortex during interphase [Almonacid et al.,^2009^]. These interphase mid1p nodes are excluded from cell tips by pom1p and passively restricted to the medial cortex [Celton-Morizur et al.,^2006^; Padte et al.,^2006^]. This pom1p-mediated tip occlusion system is also important for controlling cell polarity [Tatebe et al.,^2008^]. The finding that a partial reduction of cdc15p activity is able to restore normal septum placement in cells lacking *mid1* and *pom1* [Huang et al.,^2007^] suggests that the tip occlusion system also inhibits cdc15p and other CR proteins to prevent septation at the cell ends. This is reminiscent of the situation in bacteria, where systems exist to prevent cytokinesis at the cell tips under normal conditions; reviewed by [Oliferenko et al.,^2009^].

The second is nuclear export of mid1p at the G2/M transition, which targets mid1p to the cortex proximal to the nucleus [Sohrmann et al.,^1996^; Paoletti and Chang,^2000^; Daga and Chang,^2005^], which is likely to be mediated by plo1p [Bähler et al.,^1998^]; Plo1p directly binds to mid1p and seems to play an important role in formation of the medial mid1p ring; the protein kinase pdk1p also contributes to this process [Bimbo et al.,^2005^]. Consistent with this, both kinases localize to SPB and the medial ring in mitosis [Bähler et al.,^1998^; Bimbo et al.,^2005^]. The third regulatory mechanism is thought to be the presence of domains formation of tightly packed ER in the medial region, which helps to retain mid1p at the CR [Zhang et al.,^2010^]. Thus, redundant mechanisms including a positive spatial cue from the centrally positioned nucleus and inhibitory signals at the cell tips contribute to medial positioning of the division plane to produce similar-sized daughter cells.

### A Time Course of CR-Assembly

**Fig. 1:**
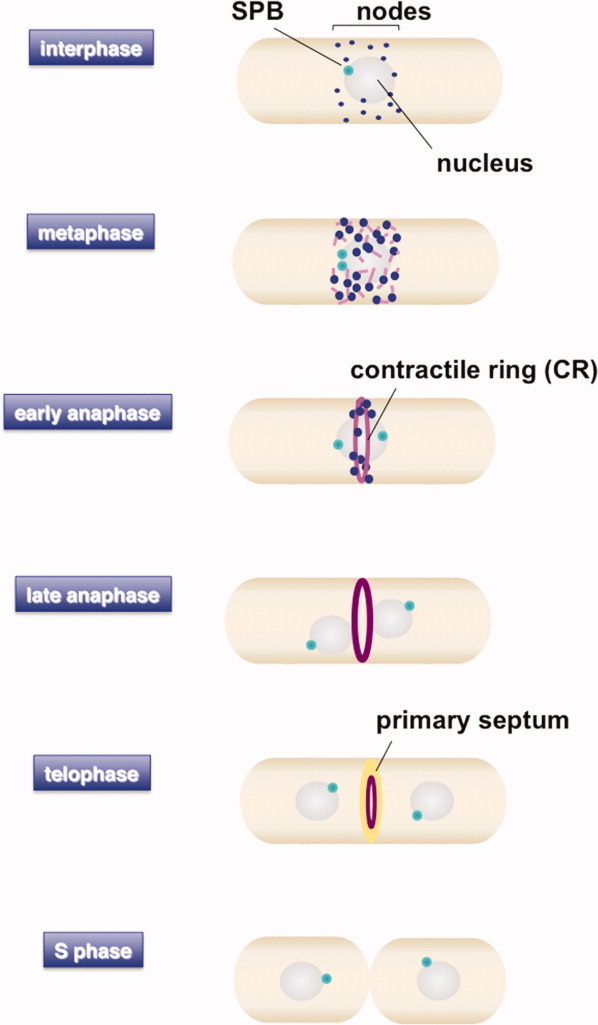
Fission yeast cytokinesis The cartoon shows the various stages of CR formation with respect to mitotic progression. See text for details.

An elegant study from the Pollard lab [Wu et al.,^2003^] used fluorescent protein tagged ring components, which had been tested as far as possible for their biological function, to examine the kinetics and timing of CR assembly. Using SPB markers, they were able to correlate this with mitotic progression. Though the behavior of many individual CR proteins had been analyzed previously, this study examined many CR markers in parallel, under similar filming and growth conditions, enabling the relative timing of their appearance at the CR to be investigated. It therefore provides an important landmark in our understanding of how the CR assembles. The process of CR assembly and cytokinesis is shown in cartoon form in Fig. 1.

When cells enter metaphase, F-actin and myosin II (myo2p) independently appear on the medial cortex and then interact with each other to form the CR [Naqvi et al.,^1999^; Motegi et al.,^2000^]. Prior to F-actin localization, accumulation of myo2p as cortical dots is induced, probably through interaction with mid1p [Motegi et al.,^2004^]. Importantly, whereas full-length myo2p will only accumulate at the medial region after onset of metaphase, a C-terminal tail region of myo2p can associate with cortical mid1p nodes before the G2/M transition, suggesting that interphase-specific auto-inhibition of localization to nodes may be an immanent property of myo2p [Motegi et al.,^2004^]. It has also been suggested that mitosis-specific dephosphorylation of Ser1444 controls the timing of association of myo2p with cortical nodes [Motegi et al.,^2004^]. Though expression of the nonphosphorylatable mutant myo2S1444A induces CR-assembly in G2-arrested cells [Motegi et al.,^2004^], a recent study casts doubt on the importance of this phosphoregulation because the same mutation does not affect the time course of myo2p accumulation into CR, and the phosphomimetic myo2S1444D mutant, can substitute for the wild-type protein without significantly affecting cytokinesis [Sladewski et al.,^2009^]. At present, it remains unclear how the auto-inhibition of myo2p is relieved at G2/M transition.

In addition to myo2p, an IQGAP-like actin-crosslinking protein rng2p, the formin cdc12p, and the F-BAR protein cdc15p are also incorporated into cortical mid1p nodes in early metaphase, independently of F-actin [Wu et al.,^2003^]. Dynamic redistribution of actin from cell tips, where actin patches are located in interphase, to the medial region occurs during formation of CR F-actin, which depends on the actin-depolymerizing factor, adf1p [Nakano and Mabuchi,^2006^]. Longitudinal F-actin cables are often seen in the middle of early metaphase cells [Arai et al.,^1998^]. However, preexisting actin cables are not essential for CR assembly, because functional CR is formed in cells lacking the cables such as *for3*-null mutant [Nakano et al.,^2002^]. Therefore, *de novo* polymerization of F-actin is the dominant pathways for CR assembly in *S. pombe*. Actin subunits depolymerized from actin patches in cell tips are bound to profilin cdc3p [Balasubramanian et al.,^1994^] and are repolymerized in the medial region of the cell, probably by cdc12p [Chang et al.,^1997^]. The cdc12p-capped barbed end of F-actin favors the use of the cdc3p-actin complex for polymerization [Kovar et al.,^2003^]. Recent biochemical and genetic analyses suggest that rng2p may be involved in actin-polymerization redundantly with cdc12p [Takaine et al.,^2009^]. Subsequent to this, myo2p dots associate with each other through interacting with F-actin and finally the CR is formed before anaphase. Thus, the timing of CR formation in *S. pombe* differs from animal cells, where assembly of the CR is induced after inactivation of CDK1 and anaphase onset. Formation of the *S. pombe* CR early in mitosis is mediated by mid1p, which acts as an organizing scaffold for the actin polymerization machinery and myo2p to the future division site. Mid1p has a C-terminal PH domain with low similarity to the metazoan cytokinesis proteins of the anillin family, which directly bind to both F-actin and myosin II [reviewed by D'Avino,^2009^]. Though mid1p may share the functional similarity with anillin, it is unclear whether these proteins have evolved from a common ancestral gene.

In *mid1* mutant cells, although CR formation is not initiated at metaphase, myo2p associates with a cable-like F-actin structure elongating from the cell cortex during anaphase, resulting in formation of an abnormally shaped CR, which is frequently displaced from the cell middle [Huang et al.,^2007^]. Therefore, mid1p-dependent events assure that *S. pombe* division is symmetrical while another system, probably SIN-mediated, supports CR-assembly. Interestingly, at least two other species of the *Schizosaccharomyces* genus, *S. japonicus* and *S. octosporus* do not have a clear ortholog of mid1p and in these organisms CR assembly is initiated during anaphase [Alfa and Hyams^1990^] (KN, unpublished data). All three species (KN, unpublished data) have a conserved anillin-related protein, named mid2p in *S. pombe*, which is required for formation of the septin ring and cell separation in *S. pombe* [Berlin et al.,^2003^; Tasto et al.,^2003^].

Flp1p/clp1p is the *S. pombe* ortholog of the conserved CDC14-family of phosphoprotein phosphatases; it is implicated in many cellular processes, and is regulated by phosphorylation and localization [Cueille et al.,^2001^; Trautmann et al.,^2001^; Wolfe et al.,^2006^]. During mitosis, it localizes to the CR by associating with mid1p and controls the dynamics of CR components such as cdc15p and myo2p [Clifford et al.,^2008^]. Flp1p/clp1p also contributes to the dephosphorylation of cdc15p, probably in cooperation with other phosphatases [Fankhauser et al.,^1995^; Wachtler et al.,^2006^; Clifford et al.,^2008^]. Flp1p/clp1p is likely to have many targets, and since it is not an essential gene, other phosphoprotein phosphatases will also doubtless regulate aspects of cytokinesis in *S. pombe*. The CR is assembled stepwise during mitosis [Wu et al.,^2003^]; when it is fully compacted at the onset of anaphase B, completion of cytokinesis requires the SIN. The following sections will describe the SIN, what it does and how it is regulated.

### The Septation Initiation Network

#### What Is the SIN?

**Fig. 2:**
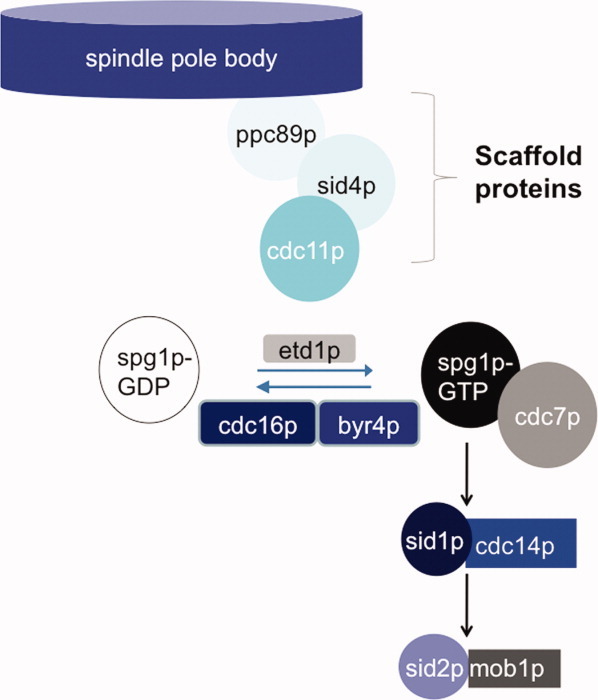
Components of the SIN The cartoon depicts the core components of the SIN and the presumed relationship between them. For the sake of clarity, nonessential regulators are not shown. See text for additional details.

The SIN is a group of protein kinases which are essential for cytokinesis. Signaling requires the activity of three protein kinases, each of which has a regulatory subunit (kinase-regulator); cdc7p-spg1p [Fankhauser and Simanis,^1994^; Schmidt et al.,^1997^; Mehta and Gould,^2006^], sid1p-cdc14p [Fankhauser and Simanis,^1993^; Guertin et al.,^2000^; Guertin and McCollum,^2001^] and sid2p-mob1p [Sparks et al.,^1999^; Hou et al.,^2000^; Salimova et al.,^2000^]. SIN signaling is modulated by the nucleotide status of the GTPase spg1p [Schmidt et al.,^1997^; Sohrmann et al.,^1998^] (Fig. 2). This is determined by the balance of spontaneous nucleotide exchange, a putative GEF, etd1p [Daga et al.,^2005^; Garcia-Cortes and McCollum,^2009^] and a GAP, cdc16p [Minet et al.,^1979^; Fankhauser et al.,^1993^], with which spg1p interacts through a scaffold, byr4p [Song et al.,^1996^; Furge et al.,^1998^; Furge et al.,^1999^]. The SIN is also activated by the mitotic regulator plo1p [Tanaka et al.,^2001^], which is the fission yeast orthologue of *Drosophila* POLO [Ohkura et al.,^1995^]. Loss of SIN signaling produces multinucleate cells, while constitutive activation of the SIN results in multiseptated cells [Minet et al.,^1979^; Fankhauser et al.,^1993^; Song et al.,^1996^]. Ectopic activation of the SIN by either overexpression of *plo1*, *cdc7*, or *spg1* promotes CR and septum formation from any stage of the cell cycle, uncoupling the usual dependency of cytokinesis upon entry into mitosis [Fankhauser and Simanis,^1994^; Ohkura et al.,^1995^; Schmidt et al.,^1997^; Guertin et al.,^2002^].

#### What Does the SIN Do?

The SIN has been implicated in assembly of contractile actin ring (CR) [Hachet and Simanis,^2008^; Huang et al.,^2008^] as well as its contraction [reviewed by Gould and Simanis,^1997^] and the subsequent synthesis of the division septum [Jin et al.,^2006^]. The CR anchor mid1p and the SIN cooperate in assembly of the CR; plo1p regulates both mid1p and the SIN, which may provide global coordination of CR assembly early in mitosis [reviewed by Roberts-Galbraith and Gould,^2008^]; see also the discussion above. The SIN has also been implicated in reformation of the interphase microtubule array from the EMTOC at the end of mitosis [Heitz et al.,^2001^], formation and/or retention of astral microtubule arrays at the SPB [Krapp et al.,^2001^], equatorial retention of the CR during mitosis [Pardo and Nurse,^2003^] and in spatial reorganization of the endocytic machinery [Gachet and Hyams,^2005^]. The SIN may regulate the morphology network, which controls cell polarity [Kanai et al.,^2005^; Mendoza et al.,^2005^; Ray et al.,^2010^], thereby contributing to the switch from polar growth to CR assembly. The SIN is also implicated in the cytokinesis checkpoint, which stabilizes a defective CR to allow time to allow it to complete its assembly [Le Goff et al.,^1999^; Liu et al.,^2000^] (see below). The kinase plo1p is also required, in conjunction with the stress-response pathway, for resumption of normal growth after stress [Petersen and Hagan,^2005^]; in most cases, the relevant phosphorylation targets in these processes have not been determined.

#### SIN Signaling Requires Association of SIN Proteins with the SPB

**Fig. 3:**
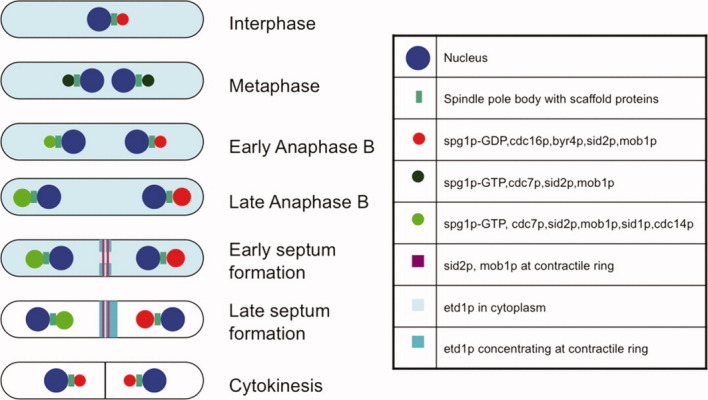
Localization of SIN proteins during interphase and mitosis The cartoon depicts the localization of the core SIN proteins during mitosis and cytokinesis. The size of the circles associated with the SPB is meant to represent the intensity of the signal that is observed. For the sake of clarity, the localization of regulatory proteins is not shown: see Lattmann et al. [2009]; Simanis [2003] for additional details.

Laser ablation of SPBs in fission yeast during mitosis suggests that at least one SPB must be intact during anaphase B for cytokinesis to occur [Magidson et al.,^2006^]. The *S. pombe* SPB is composed of cytoplasmic and nuclear components which are separated by the nuclear envelope and connected by fine striations [Ding et al.,^1997^]. The duplication of the SPB appears to be conservative [Ding et al.,^1997^; Grallert et al.,^2004^], generating “old” and “new” SPBs that can be distinguished using slow-folding RFP-tagged proteins [Grallert et al.,^2004^]. Studies of the localization of SIN proteins indicates that association with the SPB at various points of the cell cycle plays an important part in regulating the SIN, and therefore in the coordination of mitosis and cytokinesis [reviewed by Simanis,^2003^; Wolfe and Gould,^2005^; Krapp and Simanis,^2008^; Lattmann et al.,^2009^]. The distribution of the SIN proteins during mitosis is shown in Fig. 3.

The SIN proteins associate with the SPB via a tripartite scaffold comprised of ppc89p, sid4p and cdc11p [Chang and Gould,^2000^; Krapp et al.,^2001^; Tomlin et al.,^2002^; Morrell et al.,^2004^; Rosenberg et al.,^2006^]. Mutants that compromise either *sid4* or *cdc11* block association of SIN proteins with the SPB, and prevent SIN signaling. Ectopic activation of the SIN in a *sid4* mutant fails to promote septum formation, indicating that SPB association of SIN proteins is important for signaling [Balasubramanian et al.,^1998^]. The scaffold proteins are seen at the SPB throughout the mitotic cycle. The requirement for the SIN in CR assembly also suggests that the SIN has a cytoplasmic role, but it is noteworthy that all of its functions seem to require the SPB-associated scaffold molecules sid4p and cdc11p [Hachet and Simanis,^2008^].

During interphase, spg1p, byr4p, and cdc16p are all observed at the SPB; cdc16p and byr4p are interdependent for localization [Sohrmann et al.,^1998^; Cerutti and Simanis,^1999^; Li et al.,^2000^]. This tripartite complex [Furge et al.,^1998^] is presumed to be inactive for signaling the initiation of septation, but whether it has any other role at the SPB in interphase is unclear. The steady state level of byr4p is influenced by its ability to bind spg1p [Krapp et al.,^2008^], which may regulate the amount of GAP present in the cell. Following CDK activation and entry into mitosis, cdc16p is removed from the SPB. In the absence of cdc16p, byr4p prevents release and hydrolysis of GTP by spg1p *in vitro* [Furge et al.,^1998^], and may thus contribute to stabilizing active spg1p in early mitosis.

Spg1p in its GTP-bound form interacts with cdc7p during mitosis [Sohrmann et al.,^1998^; Mehta and Gould,^2006^], and is observed initially on both SPBs, where low levels of byr4p are also present [Cerutti and Simanis,^1999^; Li et al.,^2000^]. Cdc7p requires spg1p to localize to the SPB [Sohrmann et al.,^1998^], but not vice-versa, provided byr4p is present [Krapp et al.,^2008^]. After the onset of anaphase B, the constellation of SIN proteins seen at the two SPBs is different. As the poles separate, the cdc7p signal at the old SPB grows fainter while the new SPB becomes brighter, reaching a maximum as the SPBs approach the cell tips [Sohrmann et al.,^1998^; Cerutti and Simanis,^1999^; Grallert et al.,^2004^; Garcia-Cortes and McCollum,^2009^]. The GAP (byr4p-cdc16p) is reconstituted first at the old SPB, and the intensity of the signals at the old SPB increase throughout anaphase [Cerutti and Simanis,^1999^; Li et al.,^2000^], mirroring the behavior of cdc7p at the new SPB. Indirect immunofluorescence has shown that byr4p becomes asymmetric before cdc7p [Cerutti and Simanis,^1999^]. There are ∼ 400 molecules of cdc7p at the SPB in late mitosis [Wu and Pollard,^2005^] and time-lapse analysis of cdc7p has shown that its removal from the new SPB correlates with closure of the CR [Garcia-Cortes and McCollum,^2009^].

The protein kinase sid1p and its regulatory subunit cdc14p appear at the new SPB only after the inactivation of CDK and onset of anaphase B [Guertin et al.,^2000^; Dischinger et al.,^2008^]. Like cdc7p, the signal increases in intensity during anaphase B spindle elongation, peaking as the SPBs approach maximal separation. Sid1p and cdc14p are interdependent for localization to the SPB, which also requires functional *spg1* and *cdc7* [Guertin et al.,^2000^]. Sid2p and mob1p are also interdependent for localization and associate with both SPBs throughout mitosis, and also with the medial region at the time of septum formation. The sid2p-mob1p complex requires functional SIN and CR components to localize to the medial region [Sparks et al.,^1999^; Hou et al.,^2000^; Salimova et al.,^2000^]. Full activation of sid2p also requires functional cdc7p [Sparks et al.,^1999^]. Sid2p and mob1p localization to the SPB is reduced in *cdc7* mutants and some alleles of *spg1* [Sparks et al.,^1999^; Hou et al.,^2000^; Salimova et al.,^2000^].

Biochemical analysis has shown that cdc16p, spg1p, mob1p, and cdc13p (the mitotic cyclin for cdc2p) all bind to the N-terminal domain of cdc11p [Morrell et al.,^2004^]. Maintaining the association of cdc7p and sid2p with the SPB in anaphase B requires etd1p function [Daga et al.,^2005^]. Studies of the localization and activity of sid2p have given rise to a model placing it downstream of cdc7p and sid1p [Sparks et al.,^1999^; Guertin et al.,^2000^]. However, given its symmetrical distribution during anaphase, it cannot be excluded that it plays different roles at the new and old SPBs. Immunoelectron microscopy has shown that in interphase, sid2p is associated with the cytoplasmic face of the SPB [Sparks et al.,^1999^]. It is not known whether the other SIN proteins also localize to the outer face of the SPB during mitosis. Taken together, these data have led to the idea that the order of action of the SIN proteins is cdc7p-spg1p, then sid1p-cdc14p, and finally sid2p-mob1p [Guertin et al.,^2000^].

#### Factors Influencing Asymmetric SIN Protein Distribution during Mitosis

The transition from the symmetric to the asymmetric configuration of the SIN and the initiation of septation requires inactivation of mitotic CDK [Yamano et al.,^1996^; He et al.,^1997^; Guertin et al.,^2000^; Chang et al.,^2001^; Dischinger et al.,^2008^]. It has been proposed that this asymmetric distribution of proteins is important to turn off the SIN and complete cytokinesis [Garcia-Cortes and McCollum,^2009^; reviewed by Lattmann et al.,^2009^]. Consistent with this, cells in which the GAP is absent, leading to permanent activation of spg1p, show a symmetric distribution of SIN proteins during anaphase [Sohrmann et al.,^1998^; Guertin et al.,^2000^], and undergo multiple rounds of septation without cleavage [Minet et al.,^1979^; Song et al.,^1996^]. The transition to the asymmetric state of the SIN is also influenced by chromosome segregation [Mayer et al.,^2006^]; cells that contain lagging chromosomes on an elongating spindle show symmetric distribution of cdc7p; the nature of the signaling pathway involved is unknown, but probably differs from that regulating the association of mad2p with the SPB [Mayer et al.,^2006^]. Mutation of either *par1*, the B′ regulatory subunit of PP2A [Jiang and Hallberg,^2001^], or the regulator of mitotic commitment *fin1* [Grallert et al.,^2004^], result in an increase of the number of cells in which cdc7p segregates symmetrically during anaphase. In cells arrested by the cytokinesis checkpoint, cdc7p remains associated with the new SPB as long as the defective CR is present in the cell [Mishra et al.,^2004^], consistent with signaling between the CR and SPB. Loss of the phosphoprotein phosphatase flp1p/clp1p affects maintenance of cdc7p at the new SPB in cytokinesis-checkpoint arrested cells [Mishra et al.,^2004^], and also impedes recruitment of sid1p to the new SPB in late anaphase [Trautmann et al.,^2001^]. Inactivation of etd1p results in premature loss of cdc7p from the SPBs in anaphase [Daga et al.,^2005^], while excess etd1p delays removal of cdc7p from the new SPB after cell separation [Garcia-Cortes and McCollum,^2009^], leading to the proposal that a feedback mechanism regulates SIN activity; reviewed by [Lattmann et al.,^2009^].

#### Regulators of the SIN

A number of putative regulators of the SIN have been identified genetically; *zfs1* and *scw1* are RNA binding proteins [Beltraminelli et al.,^1999^; Karagiannis et al.,^2002^; Jin and McCollum,^2003^; Cuthbertson et al.,^2008^], but the RNAs involved in SIN regulation remain undefined. The nucleolar protein dnt1p [Jin et al.,^2007^] and the phosphatases PP2A [Jiang and Hallberg,^2001^; Le Goff et al.,^2001^] and flp1p/clp1p [Cueille et al.,^2001^; Trautmann et al.,^2001^] have also been identified as regulators of the SIN, though their targets remain unknown. Fully active cdc2p inhibits the SIN early in mitosis and its inactivation is required for septum formation [Yamano et al.,^1996^; He et al.,^1997^; Chang et al.,^2001^; Dischinger et al.,^2008^]; the association of cdc13p with cdc11p may facilitate this regulation [Morrell et al.,^2004^]. Furthermore, cdc2p and the SIN may cooperate to regulate septation in interphase [Cerutti and Simanis,^1999^]. Proteolysis also plays a role in regulating the SIN; resetting the SIN involves the APC/C subunit nuc2p [Chew and Balasubramanian,^2008^] and elimination of etd1p [Daga et al.,^2005^; Garcia-Cortes and McCollum,^2009^]. The putative ubiquitin ligase dma1p is an inhibitor of the SIN which is required to prevent septum formation in mitotically arrested cells [Murone and Simanis,^1996^]; it influences recruitment of plo1p to the SPB and its ubiquitin ligase domain is required for its function [Guertin et al.,^2002^]. The protein kinase fin1p requires the SIN for its localization to the SPB, and it modulates SIN signaling [Grallert et al.,^2004^], which may point to the existence of feedback regulation of the SIN. In this context it is also noteworthy that plo1p is recruited to the SPB prematurely in a *cdc7* mutant at the permissive temperature [Mulvihill et al.,^1999^]. Finally, it has been proposed that to coordinate mitotic progression and cytokinesis, the γ-tubulin complex inhibits the SIN until mitotic CDK is inactivated [Vardy et al.,^2002^].

#### SIN-Related Proteins in Other Organisms

The biological counterpart of the *S. pombe* SIN in another yeast, *Saccharomyces cerevisiae*, is called the mitotic exit network (MEN). In addition to a role in cytokinesis, the MEN is required for the inactivation of CDK, and exit from the mitotic state into G1; for reviews, see [Burke,^2009^; Rock and Amon,^2009^]. Many components are conserved structurally (and in some cases, also to the level of functional cross-complementation) between the MEN and SIN [for details see the following reviews: Bardin and Amon,^2001^; Simanis,^2003^; Stegmeier and Amon,^2004^]. Some components of the SIN have recognisable counterparts in higher eukaryotes; for example, the SIN scaffold cdc11p shares a common domain with centriolin, which is required for cytokinesis [Gromley et al.,^2003^; Gromley et al.,^2005^]. The kinase sid2p-mob1p is a member of the NDR kinase family, which is implicated in growth control and cytokinesis in multicellular eukaryotes as part of the Salvador-Warts-Hippo tumor suppressor network. In this context, it seems to function by inhibiting gene expression programs; it is unclear whether the SIN has any role in regulating gene expression [reviewed by Harvey and Tapon,^2007^; Matallanas et al.,^2008^; Hergovich and Hemmings,^2009^; Zhang et al.,^2009^].

#### A Checkpoint in CR Assembly

As mentioned above, the SIN seems to be important for stabilizing the CR after onset of anaphase and to sustain assembly of CR components until cytokinesis is finished. The SIN and flp1p/clp1p, are both implicated in a cytokinesis checkpoint, which blocks the next round of mitosis in response to perturbed assembly of the CR [Le Goff et al.,^1999^; Cueille et al.,^2001^; Trautmann et al.,^2001^; Mishra et al.,^2004^]. Flp1p/clp1p is sequestered in the nucleolus during interphase and is released to the nucleoplasm and then the cytoplasm during mitosis. The core SIN kinase sid2p phosphorylates the flp1p/clp1p to keep it in the cytoplasm, which contributes to the function of the cytokinesis checkpoint [Chen et al.,2008a].

### Autonomous Assembly of the Cytokinetic Machinery

Overexpression of C-terminal truncated cdc12p, probably corresponding to a dominant active form lacking the auto-inhibitory domain, induces cdc15p- and CR-dependent cytokinesis even in interphase cells [Yonetani and Chang,^2010^]. Overexpression of cdc15p is able to induce actin rearrangements to the medial region in G2-arrested cells. Cdc15p may be negatively regulated by phosphorylation in interphase [Fankhauser et al.,^1995^]. Cdc15p can self-assemble and may interact directly with the plasma membrane through its F-BAR domain [Roberts-Galbraith et al.,^2010^]. Dephosphorylation of cdc15p promotes its oligomerization and increases its affinity for one its binding partners, cdc12p [Roberts-Galbraith et al.,^2010^]. In view of the fact that cdc12p physically associates with cdc15p [Carnahan and Gould,^2003^], high doses of these proteins may be possible to induce actin-polymerization beneath cell cortex and ectopic CR assembly bypassing mitosis-specific regulation including phosphorylation. Ectopic activation of the SIN in interphase will also promote CR assembly and septum formation [Schmidt et al.,^1997^], bypassing the mid1p spatial cue [Hachet and Simanis,^2008^].

Though whole genome-based screening shows that more than 200 proteins are localized to the division site in *S. pombe*, only a small subset of these can trigger reorganization of F-actin, CR formation and septation when their activity is altered [Matsuyama et al.,^2006^; reviewed by Bathe and Chang,^2010^]. These may define rate-limiting steps or control points in CR assembly analogous to the pivotal role played by the rhoA GTPase in animal-cell cytokinesis (see below).

## Septum Formation and Cell Cleavage

After the completion of nuclear division, the CR constricts, which is followed by the primary septum synthesis. At present, it is unclear whether CR constriction is an active, motor-driven process, or whether it occurs passively, as the septum is deposited behind it. Previous studies have shown the following: Mutant cells that are unable to assemble a coherent CR deposit septum materials at the cell cortex [Streiblova et al.,^1984^; Marks et al.,^1992^], which suggests that the septum cannot close without constriction of the CR. In a spheroplast, where cell wall synthesis is impaired the CR does not constrict [Jochová et al.,^1991^], though it should be noted that it is not known whether all the required proteins are present in these CRs. Mutant cells that lack bgs1p/cps1p, the β-glucan synthase required for synthesis of the division septum, retain the CR with no constriction for several hours at the restrictive temperature [Liu et al.,^2000^]. This may indicate that septum synthesis is required for CR constriction; however, since this arrest is maintained by a checkpoint [Le Goff et al.,^1999^; Liu et al.,^2000^] and the mechanisms underpinning the checkpoint are incompletely understood, other interpretations are possible. Furthermore, the mechanism by which expansion of plasma membrane occurs at the division site remains poorly understood. Although exocytotic machineries such as the exocyst complex and the presumed vesicle transporter myo52p (myosin V) accumulate at the division site, neither CR constriction nor septum formation is interrupted in mutants lacking these functions [Motegi et al.,^2002^; Wang et al.,^2002^; Mulvihill et al.,^2006^]. However, the Golgi-mediated secretory pathway seems to be required for medial localization of bgs1p/cps1p [Liu et al.,^2002^]. Future studies should determine whether membrane ingression, CR constriction and septation are actively coordinated.

As the primary septum is synthesized, actin patches accumulate around the region of septation. In *S. pombe*, the Arp2/3-complex is not required for CR assembly [Wu et al.,^2006^], but is essential for formation of actin patches [McCollum et al.,^1996^]. Loss of the Arp2/3-complex function reduces the rate of CR constriction [Wu et al.,^2006^] without impairing the deposition of septal material [McCollum et al.,^1996^]. Since actin patches are the structure responsible for endocytosis [Gachet and Hyams,^2005^], it is possible that endocytosis is involved in membrane ingression at the division site and/or septation. This idea is supported by findings that mutants defective in endocytosis frequently form an abnormal septum [Castagnetti et al.,^2005^; Ge et al.,^2005^]. Interestingly, it has recently been demonstrated that endocytosis restricts the membrane domain required for localization of the exocytosis machinery to the division plane during cytokinesis of the higher plant *Araidopsis thaliana* [Boutte et al.,^2010^]. Moreover, Rab11-dependent membrane recycling between the plasma membrane and endosomes seems to recruit signaling molecules inducing furrow ingression at the cleavage site in the *Drosophila* early embryo [Cao et al.,^2008^]. Coincidently, in *S. pombe* cells the Rab11-homolog ypt3p localizes to the medial region during cytokinesis, dependent on an intact actin cytoskeleton [Cheng et al.,^2002^]. Therefore, it is possible that endocytosis and membrane recycling may also function in cell cleavage in this *S. pombe*.

The primary septum is composed of linear chains of 1,3-β-glucan [Humbel et al.,^2001^]. After completion of the primary septum synthesis, the secondary septum, which becomes the cell wall of the new end of the daughter cell, is synthesized on both sides of the primary septum. Finally, cell separation is induced by enzymatic degradation of the primary septum by glucanases such as the endo β-1,3-glucanase, eng1p, and endo-α-glucanase, agn1p [Martin-Cuadrado et al.,^2005^]. Mutations defective in either glucanase activity or extracellular secretion of the enzymes result in highly elongated and multicompartmented cells (chains of cells, each containing one nucleus, separated by an uncleaved septum). The transcription factor ace2p controls a late-cell-cycle specific gene expression program, which is required for cell separation; deletion of *ace2* produces multicompartmented cells [Alonso-Nunez et al.,^2005^; Petit et al.,^2005^].

## Mechanics and Dynamics of the CR in Fission Yeast

In this section, we will discuss the assembly, constriction and disassembly of the CR, with particular emphasis on the mechanical and structural aspects of these processes. We will also compare the properties of the *S. pombe* CR with those of mammalian cells, and speculate upon possible mechanisms for its assembly and constriction. We will begin with a brief review of the mechanics of cytokinesis in mammalian cells.

### General Overview of Cytokinesis in Eukaryotic Cells

In animal cells, cytokinesis is brought about by membrane ingression, which has been named the cleavage furrow (CF). The CF is thought to be induced by geometrical asymmetry of the cortical tension in the cell. Several mechanisms for CF formation have been proposed [reviewed by Wang,^2005^]; currently, the most favored one is the purse-string model [reviewed by Mabuchi and Itoh,^1992^; Noguchi et al.,^2001^; Pollard,^2010^]. The equatorial CR is composed of F-actin filaments and myosin II, and their interaction produces the force required for membrane ingression at the site of division. The concentration of F-actin at the CR seems to be particularly evident in nonmotile cells such as early embryos or yeasts. Though the F-actin at the CR is often parallel to the equator of the cell, there are also examples where cortical F-actin or myosin II are observed perpendicular to the division plane in some dividing cells [Fishkind and Wang,^1993^; DeBiasio et al.,^1996^; Oegema et al.,^2000^; Murthy and Wadsworth,^2005^; Chen et al.,2008b]. According to the purse-string model, these cortical actomyosin filaments should not contribute to constriction of the ring. Moreover, there are some cell types, especially those which are adherent to a substrate, which are able to divide without using a CR [Neujahr et al.,^1997^; O'Connell et al.,^2001^; Kanada et al.,^2005^]; in these cases, it seems that coordination of the compression of the equatorial cortex and cortical expansion at the cell poles is sufficient to induce a CF. Globally, it is likely that these mechanisms are not mutually exclusive but interdependent, and the extent to which each of them contributes to CF ingression differs according to the cell type. Thus, though cytokinesis is a fundamental cellular event, the mechanism that brings it about in any given cell-type will be a mixture of conserved and cell-type-specific features.

In animal cells, the central spindle and overlap region of astral microtubules (MTs), which extend from opposite spindle poles, promote CR assembly at telophase. Centralspindlin, a protein complex consisting of MKLP1, a kinesin-6 dimer, and Rho-family GTPase-activating protein (RhoGAP) subunits, plays the central role in this system [Mishima et al.,^2002^]. Cell cycle-dependent kinase 1 (CDK1), which promotes mitotic events such as centriole duplication, chromosome condensation, and spindle formation, phosphorylates MKLP1 during metaphase, which prevents it from interacting with MTs [Mishima et al.,^2004^]. After CDK1 is inactivated at the onset of anaphase, the protein-phosphatase CDC14 dephosphorylates MKLP1, and Centralspindlin moves on MTs and accumulates at their equatorial plus-ends. The POLO-like kinase, which regulates multiple mitotic events in addition to cytokinesis, induces association of ECT2, a guanine nucleotide-exchange factor for Rho GTPase, with Centralspindlin and localizes this protein complex to the equatorial region of the anaphase cell [reviewed by Petronczki et al.,^2008^; Wolfe et al.,^2009^]. As a result, Rho is specifically activated there and induces CR formation by promoting actin-polymerization and activation of myosin II via effectors such as formin and Rho-kinase. Furthermore, in the equatorial region of *Caenorhabditis elegans* oocyte Centralspindlin also reduces Rac GTPase-activity, which inhibits the formation of an Arp2/3-complex-dependent branched actin network and thereby ensures formation of the formin-dependent CR, which is composed of straight F-actin [Canman et al.,^2008^]. In addition to this, the tips of astral MTs which contact the cell poles relax cortical tension at the polar regions, which leads to a relative increase in equatorial contractility in *C. elegans* oocyte [reviewed by Werner and Glotzer,^2008^].

Cytokinesis is accomplished by the scission of a “bridge” connecting the daughter cells. Dynamic reorganization of membranes including scrambling of outer and inner sides of plasma membrane, SNARE–mediated vesicle fusions, and membrane scission by ESCRT complex is required for this step [reviewed by Prekeris and Gould,^2008^]. Vesicle fusion is particularly important for cytokinesis in higher plant cells where formation of the cell plate progresses centrifugally [reviewed by Jurgens,^2005^; Otegui et al.,^2005^; Dhonukshe et al.,^2007^]. Interestingly, an archaeal ortholog of Vps4, a protein stimulating ESCRT-mediated membrane scission, is required for cell division in the crenarchaeon *Sulfolobus acidocaldarius* [Lindas et al.,^2008^]. Therefore, it is possible that some molecular systems in cytokinesis may be conserved across evolutionary kingdoms; alternatively, their mechanistic similarity may reflect convergent evolution. Future study of the molecular basis for cytokinesis in various cells and organisms will doubtless help to answer this question.

### Assembly and Arrangement of CR F-Actin in Fission Yeast

In this section, we will address two questions: first, what is the origin of CR F-actin? Second, is the CR formed by a spot/leading cable mechanism, or from a band of nodes? Considerable effort has been devoted to analysis of CR assembly [Vavylonis et al.,^2008^; Yonetani et al.,^2008^; Coffman et al.,^2009^]; reviewed by [Mishra and Oliferenko,^2008^; Roberts-Galbraith and Gould,^2008^; Bathe and Chang,^2010^; Pollard and Wu,^2010^]. These studies have given rise to two models for the initial assembly of CR F-actin in fission yeast. One is the node model, which assumes that short F-actin filaments are nucleated in random directions from about 65 nodes, each of which contains two cdc12p dimers [Wu and Pollard,^2005^], and then condensed into a continuous ring by a search, capture, pull and release (SCPR) mechanism that is driven by myosin II motor activity [Wu et al.,^2006^; Pollard,^2008^; Vavylonis et al.,^2008^; Pollard and Wu,^2010^]. The other is the spot model, which proposes that F-actin cables emanating from a single or several spot(s) of clustered cdc12p encircle the cell equator to form a ring [Chang et al.,^1997^; Chang,^1999^; Arai and Mabuchi,^2002^; Wong et al.,^2002^; Carnahan and Gould,^2003^; Motegi et al.,^2004^; Kamasaki et al.,^2007^]. These two models are consistent in that they agree that CR F-actin is polymerized *de novo* at mitosis (and does not arise from conversion of interphase actin cables or patches), but they are differ significantly with regard to how CR F-actin is formed: from dozens of cdc12p nodes or from a small number of cdc12p spots. Recent studies have shown that the cdc12p spot formation depends on its FH3 domain and the actin-bundling protein ain1p, and is dispensable for CR assembly [Yonetani et al.,^2008^; Coffman et al.,^2009^]. Thus, it is likely that recruitment of cdc12p to the medial region and concomitant initial CR F-actin assembly may be mediated through multiple distinct mechanisms operating individually or in parallel, reminiscent of the situation in mammalian cells, where multiple redundant mechanisms also operate (see above). Coffman et al. found that cdc12p appears in, at least, four distinct forms: speckle (one cdc12p dimer), node (two cdc12p dimers), spot (a probable cluster of cdc12p dimers) and ring [Coffman et al.,^2009^]. It is possible that the abundance of each form is dictated by the extent of aggregation of cdc12p. Analysis of how oligomerization of cdc12p affects its actin polymerization activity may help to elucidate the way in which multiple modes of initial CR F-actin assembly vary.

#### Comparison of S. pombe CR function with mammalian cytokinesis

At least two nonexclusive mechanisms seem to be involved in recruitment of actin to the equatorial region during cytokinesis in animal cells: cortical flow and *de novo* assembly. The cortical flow of preformed F-actin is supported by direct observations of flux of fluorescently labeled actin [Cao and Wang,^1990^; Chen et al.,2008b] or GFP-actin [Zhou and Wang,^2008^] toward the CF. Although mechanism of the cortical flow remains elusive, the actin flux is suppressed by myosin II specific inhibitor blebbistatin, suggesting that myosin II motor is involved in these processes. The *de novo* assembly of CR F-actin was observed as accumulation of actin at the equator of blebbistatin-treated mitotic cells where the cortical flow is abolished [Guha et al.,^2005^; Murthy and Wadsworth,^2005^; Zhou and Wang,^2008^]. Involvement of formins that fascilitate actin assembly in cytokinesis also supports this hypothesis [Severson et al.,^2002^; Watanabe et al.,^2008^].

#### Structure and properties of CR F-actin bundles

It is generally accepted that the CR is comprised mainly of actin and myosin II. However, their precise configuration in the ring remains undetermined. EM observations in several dividing cells have revealed that CR F-actin generally aligns parallel with the equator beneath the plasma membrane at the division site [Schroeder,^1972^; Sanger and Sanger,^1980^; Yasuda et al.,^1980^; Maupin and Pollard,^1986^; Kanbe et al.,^1989^; Kamasaki et al.,^2007^]. These filaments are often composed of many small bundles in series, each of which has an average length of 0.6 μm in *S. pombe* cells. The CR actin filaments have mixed polarities, and interdigitating myosin-like thick filaments between them were also observed in animal cells [Sanger and Sanger,^1980^; Maupin and Pollard,^1986^]. Based on these observations, the purse-string model is favored as the mechanism for constriction of CR [Schroeder,^1972^; Mabuchi and Itoh,^1992^; Satterwhite and Pollard,^1992^]. This model assumes that sliding of anti-parallel F-actin via interaction with myosin II filaments shortens the unitary bundles of CR, like the contraction of muscle sarcomeres, and hence drive shrinkage of the ring. The validity of this model in *S. pombe* is discussed below.

As mentioned above, cortical F-actin filaments oriented perpendicular to CR, which would not be involved in CR constriction according to the purse-string model, are often detectable. Filaments with this orientation have escaped detection by EM observation. Given the high turnover rates of actin and myosin II in CR, this perpendicular F-actin may also be dynamic, probably more than CR F-actin, and hence may be difficult to preserve during severe fixation. Functional dissection of these filaments might substantiate alternative mechanisms for cortical ingression during cytokinesis [Wang,^2005^; Eggert et al.,^2006^]. Along similar lines, it should also be noted that a measurable amount of short F-actin filaments orthogonally crossing the CR F-actin were detected in dividing fission yeast cells [Kamasaki et al.,^2007^].

Some actin-binding proteins (ABPs) localized selectively at CR are thought to characterize the special properties of CR F-actin bundles. The actin-bundling protein α-actinin localizes to the CF in dividing NRK cells and is involved in accumulation of CR F-actin [Mukhina et al.,^2007^]; Another actin-bundling protein EPLIN also locates to the CF during cytokinesis in HeLa cells and regulates local accumulation of active myosin II [Chircop et al.,^2009^]; IQGAP proteins accumulate in CF regions of dividing sea urchin eggs [Nishimura and Mabuchi,^2003^] and mouse oocyte [Bielak-Zmijewska et al.,^2008^] and might be involved in cytokinesis. Anillin also bundles F-actin and localizes to CR in *Drosophila* and HeLa cells [Field and Alberts,^1995^; Straight et al.,^2005^]. In fission yeast cells, actin-bundling proteins including α-actinin ain1p, fimbrin fim1p, the calponin-like stg1p, and the IQGAP rng2p localize to the CR and affect the organization of CR F-actin [Eng et al.,^1998^; Nakano et al.,^2001^; Wu et al.,^2003^; Nakano et al.,^2005^; Takaine et al.,^2009^].

It is noteworthy that the equatorial accumulation of F-actin is not always evident in dividing cells, whereas ABPs or myosin II are clearly detectable in most cases [e.g., Neujahr et al.,^1997^; Foe and von Dassow,^2008^; Watanabe et al.,^2008^]. In fission yeast, the N-terminal head (actin-binding) domain of myosin II localizes by itself specifically to CR F-actin, but not to F-actin patches or cables [Lord et al.,^2005^]. Moreover, overexpression of rng2p induces formation of F-actin bundles associated with myo2p (MT and KN, unpublished data). Therefore, myosin II proteins may be tethered to the CR F-actin through the specific protein interactions, not simply because of the high concentration of F-actin. Although anillin interacts directly with myosin II, its depletion barely affects the CR localization of myosin II [Straight et al.,^2005^]. Similarly, even in the absence of mid1p, myo2p localizes only to CR F-actin in fission yeast [Motegi et al.,^2004^]. Biochemical examination of the interactions between myosin II and F-actin network or bundles induced by these proteins should elucidate the nature of specific targeting of myosin II to CR F-actin.

It also remains possible that myosin II contributes to organization of CR F-actin bundles. Myosin II is implicated in rearrangement of the actin cytoskeleton in protruding lamellae of fibroblast cells [Verkhovsky et al.,^1995^]. Myosin II filaments are also involved in assembly of stress fibers [Hotulainen and Lappalainen,^2006^] and confer contractility upon them. In particular, it is of great interest that active myosin II motors induce reorganization of F-actin into various patterns only in collaboration with actin-bundling proteins [Backouche et al.,^2006^]. In these systems myosin II is thought to act as an active F-actin crosslinkers, which can generate force between filaments using the energy of ATP hydrolysis, whereas so-called actin-bundling proteins act as passive crosslinkers and do not produce any work. Thus self-organization of F-actin induced by both active and passive crosslinkers might contribute to CR formation in fission yeast, which is partially conceptualized in the search, capture, pull and release (SCPR) model. Future studies will doubtless test this hypothesis.

### Constriction and Disassembly of CR and the Functions of Myosin II in CR Constriction

In the purse-string hypothesis, myosin II motors are presumed to generate a force driving CR constriction via interaction with CR F-actin. Inhibition of the ATPase activity of myosin II using blebbistatin blocks cytokinesis in mammalian cells without affecting CR F-actin assembly [Straight et al.,^2003^]. Interestingly, studies using blebbistatin also revealed that myosin II is involved in turnover of CR F-actin [Guha et al.,^2005^; Murthy and Wadsworth,^2005^].

*S. pombe* has two myosin II isoforms, myo2p and myo3p (also called myp2p), both of which localize to the CR [Bezanilla and Pollard,^2000^]. Myo2p is essential for cytokinesis, while myo3p is required for growth under stress conditions. Localization of the myosins to cortical nodes and the CR has been analyzed extensively [Naqvi et al.,^1999^; Motegi et al.,^2000^; Mulvihill et al.,^2001^; Wu et al.,^2003^; Motegi et al.,^2004^; Martin-Garcia and Valdivieso,^2006^; Sladewski et al.,^2009^]. The precise function of myosin II in *S. pombe* cytokinesis remains unclear. F-actin accumulates at the division site in *myo2*-null cells but fails to form a ring [Kitayama et al.,^1997^]. Biochemical experiments using purified myo2p revealed that a G345R mutation (the temperature-sensitive *myo2-E1* allele), significantly reduces the actin-binding and motor activity of myo2p [Lord and Pollard,^2004^]. In the *myo2-E1* mutant cells, node condensation into a ring abolished at restrictive temperature and is delayed at the permissive temperature [Coffman et al.,^2009^]. Moreover, CRs in *myo2-E1* cells constrict twofold slower than those in wild-type cells [Stark et al.,^2010^]. These observations are consistent with the SCPR model in that myosin II functions both in assembly of the F-actin ring and contraction of the CR. Myo3p does not appear as an equatorial band of nodes in early mitosis and joins the CR after myo2p has formed a ring [Wu et al.,^2003^]; this may indicate that myo3p plays an important role in CR constriction. Alternatively, since myo3p associates physically with chitin-synthase, it may contribute to CR closure via septum formation [Martin-Garcia and Valdivieso,^2006^]. Since a null-mutation of *myo3* exacerbates the phenotype of *myo2-E1* cells, the two myosin-IIs seem to share overlapping functions in cytokinesis [Motegi et al.,^2000^].

Folding of the myosin head domain requires a co-chaperone of the (UNC-45/CRO1/She4p) family of proteins. In *C. elegans*, UNC-45 colocalizes with myosin II during cytokinesis in the early embryos [Barral et al.,^2002^; Kachur et al.,^2004^] and is required for assembling thick filaments of myosin II in sarcomeres [reviewed by Kachur and Pilgrim,^2008^]. *S. pombe* rng3p belongs to the same UCS -family and associates with *myo2* and other four myosin proteins cotranslationally [Amorim and Mata,^2009^]. Rng3p is involved in CR formation cooperatively with myo2p [Wong et al.,^2000^] and promotes motor activity of myo2p for F-actin [Lord and Pollard,^2004^; Lord et al.,^2008^]. Rng3p localizes to the CR just before CR constriction in wild-type cells [Lord and Pollard,^2004^], suggesting that rng3p may induce myo2p-dependent CR constriction. Interestingly, rng3p is concentrated in nodes at metaphase in *myo2-E1* cells [Lord et al.,^2008^].

#### Comparison of Myosin Function in S. pombe and Mammalian Cytokinesis

One of the observable characteristics of myosin II molecules is to assemble into bipolar thick filaments through their tails [Kaminer and Bell,^1966^]. When considering the role of myosin II in cytokinesis, its filamentogenesis should be examined because the bipolar filaments are indispensable prerequisite for CR constriction according to the purse-string model. In dividing *Dictyostelium* or *Drosophila* S2 cells, accumulation of individual myosin-II filaments at the equatorial region has been visualized as small rods less than 1 μm in length by fluorescence microscopy [Yumura and Fukui,^1985^; Yumura et al.,^2008^; Vale et al.,^2009^]. To date, no such myosin-II-containing rod-shaped structures have been observed in fission yeast cells. Biochemical studies have revealed that both myo2p purified from fission yeast cells and bacterially expressed myo3p tail are insoluble at physiological salt concentrations and become soluble in high salts like common myosin-IIs [Bezanilla and Pollard,^2000^; Lord and Pollard,^2004^]. Quantitative microscopic analysis estimated that about 40 molecules of myo2p are present in each cortical node, so the local concentration of myo2p in CR would be 20 μM [Wu and Pollard,^2005^], which both seems to be enough to form thick filaments. However, it is still unknown whether these myosin-IIs form ordered bipolar filaments in each node or in CR. In this context, it will be important to investigate whether the rng3p has an ability to induce oligomerization of myo2p.

### Functions of Actin-Depolymerizing Factor in Cytokinesis

FRAP analysis has shown that CR F-actin and cortical F-actin turn over rapidly both in fission yeast and mammalian cells [Pelham and Chang,^2002^; Guha et al.,^2005^; Murthy and Wadsworth,^2005^]. The actin-depolymerizing factor (ADF)/cofilin-family (hereafter refer to as ADF) proteins facilitate depolymerization and severing of actin filaments and hence play a key role in regulation of F-actin dynamics in various motile processes; reviewed by [Pantaloni et al.,^2001^]. In some animal cells, ADF proteins are required for completion of cytokinesis, but not for assembly of the CR [Gunsalus et al.,^1995^; Somma et al.,^2002^; Kaji et al.,^2003^; Ono et al.,^2003^]. It is likely that these ADFs contribute to turnover of CR F-actin or its active disassembly.

In *S. pombe* cells, the sole ADF, named as adf1p, localizes to the CR and is essential for accumulation of F-actin at the division site during cytokinesis [Nakano and Mabuchi,^2006^]. This is consistent with the observation that *Xenopus* ADF/cofilin, XAC, localizes to the leading edge of the CF in a fertilized egg and also participates in its formation [Abe et al.,^1996^]. Complementation-assays in fission yeast using mutant ADFs suggest that the actin-severing activity of adf1p may primarily contribute to its function in cytokinesis rather than its actin-depolymerizing activity. Actin-severing increases number of filament ends, which is required to induce dynamic turnover of actin subunits, since internal subunits are never exchanged in actin filaments. Andrianantoandro and Pollard, however, revealed that high concentration of adf1p nucleates actin polymerization whereas it effectively severs F-actin at lower concentrations [Andrianantoandro and Pollard,^2006^]. Thus, in fission yeast, adf1p would promote turnover of CR F-actin by a combination of its actin-severing and -nucleation activities. Another F-BAR protein, imp2p and a paxillin-related protein, pxl1p may also be implicated in CR disassembly or constriction [Demeter and Sazer,^1998^; Ge et al.,^2005^; Pinar et al.,^2008^], though their mechanism of action in this context remains unclear.

### Possible Functions of CR and Mechanisms for Its Constriction

It is unequivocal that in dividing animal cells net inward forces are generated along the equator, whether by the CR or by other mechanisms, and they drive the constriction of the medial cortex [Rappaport,^1967^; Hiramoto,^1975^]. On the other hand, contribution of CR to the generation of contractile force is elusive in fission yeast as discussed above. In the budding yeast, *S. cerevisiae*, the CR is not essential for cytokinesis, and septation alone manages to carry out the closure of bud neck [Bi et al.,^1998^]. The SIN pathway is not only required for localization of β-1,3-glucan synthase, bgs1p, at the division plane for formation of the primary septum [Liu et al.,^2002^], but is also essential for maturation of CR [Hachet and Simanis,^2008^]. Hyperactivation of the SIN pathway induces ectopic F-actin ring formation [Schmidt et al.,^1997^]. Taken together, CR constriction and septation would be interdependent in both mechanical and signaling aspects, and it may be difficult to distinguish separately their functions in cytokinesis. We believe, however, that fission yeast CR shares enough functional molecular bases with those of animal cells to serve as a model system.

Whatever the actual function of CR constriction is, its mechanism(s) is still an open question. As discussed above, the purse-string model should be challenged by examining whether myo2p assembles into minifilaments *in vitro*. Fine and time-resolved EM observation of CR and the medial region of dividing fission yeast cells may also help to address this issue. An attractive model for generation of the contractile force has been proposed, which posits that polymerization and depolymerization of CR F-actin can produce mechanical force for the ring contraction in the presence of end-tracking crosslinkers even without myosin II motors [Zumdieck et al.,^2007^]. In this model, tracking of a depolymerizing filament end by the crosslinker, which also binds to the second filament, is harnessed to generate sliding force between the two filaments. At present, the model remains to be validated, but it clearly seems worth pursuing, given the general and critical importance of both ADFs and formin proteins in cytokinesis.

## Conclusions

The fission yeast *Schizosaccharomyces pombe* has proved to be an informative model for the study of cytokinesis and its coordination with mitosis. Many of the structural components required for cytokinesis are well conserved through evolution, so the fission yeast provides an excellent background in which to test the *in vivo* effects of defined mutations; when proteins are involved in multiple processes, hypomorphic alleles may permit separation of these functions. Though we have learnt a great deal about how cytokinesis occurs in *S. pombe* through a judicious mixture of cell biology, genetics and biochemical analysis, it is clear from the foregoing discussion that much remains to be done. Scientifically speaking, the next few years should be very exciting!

## References

[b1] Abe H, Obinata T, Minamide LS, Bamburg JR (1996). *Xenopus laevis* actin-depolymerizing factor/cofila phosphorylation-regulated protein essential for development. J Cell Biol.

[b2] Alfa CE, Hyams JS (1990). Distribution of tubulin and actin through the cell division cycle of the fission yeast *Schizosaccharomyces japonicus* var. versatilis: a comparison with *Schizosaccharomyces pombe*. J Cell Sci.

[b3] Almonacid M, Moseley JB, Janvore J, Mayeux A, Fraisier V, Nurse P, Paoletti A (2009). Spatial control of cytokinesis by Cdr2 kinase and Mid1/anillin nuclear export. Curr Biol.

[b4] Alonso-Nunez ML, An H, Martin-Cuadrado AB, Mehta S, Petit C, Sipiczki M, del Rey F, Gould KL, de Aldana CR (2005). Ace2p controls the expression of genes required for cell separation in *Schizosaccharomyces pombe*. Mol Biol Cell.

[b5] Amorim MJ, Mata J (2009). Rng3, a member of the UCS family of myosin co-chaperones, associates with myosin heavy chains cotranslationally. EMBO Rep.

[b6] Andrianantoandro E, Pollard TD (2006). Mechanism of actin filament turnover by severing and nucleation at different concentrations of ADF/cofilin. Mol Cell.

[b7] Arai R, Mabuchi I (2002). F-actin ring formation and the role of F-actin cables in the fission yeast *Schizosaccharomyces pombe*. J Cell Sci.

[b8] Arai R, Nakano K, Mabuchi I (1998). Subcellular localization and possible function of actin, tropomyosin and actin-related protein 3 (Arp3) in the fission yeast *Schizosaccharomyces pombe*. Eur J Cell Biol.

[b9] Backouche F, Haviv L, Groswasser D, Bernheim-Groswasser A (2006). Active gels: dynamics of patterning and self-organization. Phys Biol.

[b10] Bähler J, Pringle JR (1998). Pom1p, a fission yeast protein kinase that provides positional information for both polarized growth and cytokinesis. Genes Dev.

[b11] Bähler J, Steever AB, Wheatley S, Wang Y, Pringle JR, Gould KL, McCollum D (1998). Role of polo kinase and Mid1p in determining the site of cell division in fission yeast. J Cell Biol.

[b12] Balasubramanian MK, Hirani BR, Burke JD, Gould KL (1994). The *Schizosaccharomyces pombe cdc3*+ gene encodes a profilin essential for cytokinesis. J Cell Biol.

[b13] Balasubramanian MK, McCollum D, Chang L, Wong KC, Naqvi NI, He X, Sazer S, Gould KL (1998). Isolation and characterization of new fission yeast cytokinesis mutants. Genetics.

[b14] Bardin AJ, Amon A (2001). MEN and Swhat's the difference?. Nat Rev Mol Cell Biol.

[b15] Barral JM, Hutagalung AH, Brinker A, Hartl FU, Epstein HF (2002). Role of the myosin assembly protein UNC-45 as a molecular chaperone for myosin. Science.

[b16] Bathe M, Chang F (2010). Cytokinesis and the contractile ring in fission yeast: towards a systems-level understanding. Trends Microbiol.

[b17] Beach D, Durkacz B, Nurse P (1982). Functionally homologous cell cycle control genes in budding and fission yeast. Nature.

[b18] Beltraminelli N, Murone M, Simanis V (1999). The *S. pombe zfs1* gene is required to prevent septation if mitotic progression is inhibited. J Cell Sci.

[b19] Berlin A, Paoletti A, Chang F (2003). Mid2p stabilizes septin rings during cytokinesis in fission yeast. J Cell Biol.

[b20] Bezanilla M, Pollard TD (2000). Myosin-II tails confer unique functions in *Schizosaccharomyces pombe*: characterization of a novel myosin-II tail. Mol Biol Cell.

[b21] Bi E, Maddox P, Lew DJ, Salmon ED, McMillan JN, Yeh E, Pringle JR (1998). Involvement of an actomyosin contractile ring in *Saccharomyces cerevisiae* cytokinesis. J Cell Biol.

[b22] Bielak-Zmijewska A, Kolano A, Szczepanska K, Maleszewski M, Borsuk E (2008). Cdc42 protein acts upstream of IQGAP1 and regulates cytokinesis in mouse oocytes and embryos. Dev Biol.

[b23] Bimbo A, Liu J, Balasubramanian MK (2005). Roles of Pdk1p, a fission yeast protein related to phosphoinositide-dependent protein kinase, in the regulation of mitosis and cytokinesis. Mol Biol Cell.

[b24] Boutte Y, Frescatada-Rosa M, Men S, Chow C, Ebine K, Gustavsson A, Johansson L, Ueda T, Moore I, Jurgens G, Grebe M (2010). Endocytosis restricts *Arabidopsis* KNOLLE syntaxin to the cell division plane during late cytokinesis. EMBO J.

[b25] Burke DJ (2009). Interpreting spatial information and regulating mitosis in response to spindle orientation. Genes Dev.

[b26] Canman JC, Lewellyn L, Laband K, Smerdon SJ, Desai A, Bowerman B, Oegema K (2008). Inhibition of Rac by the GAP activity of centralspindlin is essential for cytokinesis. Science.

[b27] Cao J, Albertson R, Riggs B, Field CM, Sullivan W (2008). Nuf, a Rab11 effector, maintains cytokinetic furrow integrity by promoting local actin polymerization. J Cell Biol.

[b28] Cao LG, Wang YL (1990). Mechanism of the formation of contractile ring in dividing cultured animal cells. II. Cortical movement of microinjected actin filaments. J Cell Biol.

[b29] Carnahan RH, Gould KL (2003). The PCH family protein, Cdc15p, recruits two F-actin nucleation pathways to coordinate cytokinetic actin ring formation in *Schizosaccharomyces pombe*. J Cell Biol.

[b30] Castagnetti S, Behrens R, Nurse P (2005). End4/Sla2 is involved in establishment of a new growth zone in *Schizosaccharomyces pombe*. J Cell Sci.

[b31] Celton-Morizur S, Racine V, Sibarita JB, Paoletti A (2006). Pom1 kinase links division plane position to cell polarity by regulating Mid1p cortical distribution. J Cell Sci.

[b32] Cerutti L, Simanis V (1999). Asymmetry of the spindle pole bodies and spg1p GAP segregation during mitosis in fission yeast. J Cell Sci.

[b33] Chang F (1999). Movement of a cytokinesis factor cdc12p to the site of cell division. Curr Biol.

[b34] Chang F, Woollard A, Nurse P (1996). Isolation and characterization of fission yeast mutants defective in the assembly and placement of the contractile actin ring. J Cell Sci.

[b35] Chang F, Drubin D, Nurse P (1997). cdc12p, a protein required for cytokinesis in fission yeast, is a component of the cell division ring and interacts with profilin. J Cell Biol.

[b36] Chang L, Gould KL (2000). Sid4p is required to localize components of the septation initiation pathway to the spindle pole body in fission yeast. Proc Natl Acad Sci USA.

[b37] Chang L, Morrell JL, Feoktistova A, Gould KL (2001). Study of cyclin proteolysis in anaphase-promoting complex [APC] mutant cells reveals the requirement for APC function in the final steps of the fission yeast septation initiation network. Mol Cell Biol.

[b38] Chen CT, Feoktistova A, Chen JS, Shim YS, Clifford DM, Gould KL, McCollum D (2008a). The SIN kinase Sid2 regulates cytoplasmic retention of the *S. pombe* Cdc14-like phosphatase Clp1. Curr Biol.

[b39] Chen W, Foss M, Tseng KF, Zhang D (2008b). Redundant mechanisms recruit actin into the contractile ring in silkworm spermatocytes. PLoS Biol.

[b40] Cheng H, Sugiura R, Wu W, Fujita M, Lu Y, Sio SO, Kawai R, Takegawa K, Shuntoh H, Kuno T (2002). Role of the Rab GTP-binding protein Ypt3 in the fission yeast exocytotic pathway and its connection to calcinurin function. Mol Cell Biol.

[b41] Chew TG, Balasubramanian MK (2008). Nuc2p, a subunit of the anaphase-promoting complex, inhibits septation initiation network following cytokinesis in fission yeast. PLoS Genet.

[b42] Chircop M, Oakes V, Graham ME, Ma MP, Smith CM, Robinson PJ, Khanna KK (2009). The actin-binding and bundling protein, EPLIN, is required for cytokinesis. Cell Cycle.

[b43] Clifford DM, Wolfe BA, Roberts-Galbraith RH, McDonald WH, Yates JR, Gould KL (2008). The Clp1/Cdc14 phosphatase contributes to the robustness of cytokinesis by association with anillin-related Mid1. J Cell Biol.

[b44] Coffman VC, Nile AH, Lee IJ, Liu H, Wu JQ (2009). Roles of formin nodes and myosin motor activity in Mid1p-dependent contractile-ring assembly during fission yeast cytokinesis. Mol Biol Cell.

[b45] Cueille N, Salimova E, Esteban V, Blanco M, Moreno S, Bueno A, Simanis V (2001). Flp1, a fission yeast orthologue of the s. cerevisiae CDC14 gene, is not required for cyclin degradation or rum1p stabilisation at the end of mitosis. J Cell Sci.

[b46] Cuthbertson BJ, Liao Y, Birnbaumer L, Blackshear PJ (2008). Characterization of zfs1 as an mRNA binding and destabilizing protein in *Schizosaccharomyces pombe*. J Biol Chem.

[b47] Daga RR, Chang F (2005). Dynamic positioning of the fission yeast cell division plane. Proc Natl Acad Sci USA.

[b48] Daga RR, Lahoz A, Munoz MJ, Moreno S, Jimenez J (2005). Etd1p is a novel protein that links the SIN cascade with cytokinesis. EMBO J.

[b49] D'Avino PP (2009). How to scaffold the contractile ring for a safe cytokinesis-lessons from Anillin-related proteins. J Cell Sci.

[b50] DeBiasio RL, LaRocca GM, Post PL, Taylor DL (1996). Myosin II transport, organization, and phosphorylation: evidence for cortical flow/solation-contraction coupling during cytokinesis and cell locomotion. Mol Biol Cell.

[b51] Demeter J, Sazer S (1998). *imp2*, a new component of the actin ring in the fission yeast *Schizosaccharomyces pombe*. J Cell Biol.

[b52] Dhonukshe P, Samaj J, Baluska F, Friml J (2007). A unifying new model of cytokinesis for the dividing plant and animal cells. Bioessays.

[b53] Ding R, West RR, Morphew DM, Oakley BR, McIntosh JR (1997). The spindle pole body of *Schizosaccharomyces pombe* enters and leaves the nuclear envelope as the cell cycle proceeds. Mol Biol Cell.

[b54] Dischinger S, Krapp A, Xie L, Paulson JR, Simanis V (2008). Chemical genetic analysis of the regulatory role of Cdc2p in the *S. pombe* septation initiation network. J Cell Sci.

[b55] Eggert US, Mitchison TJ, Field CM (2006). Animal cytokinesis: from parts list to mechanisms. Annu Rev Biochem.

[b56] Eng K, Naqvi NI, Wong KC, Balasubramanian MK (1998). Rng2p, a protein required for cytokinesis in fission yeast, is a component of the actomyosin ring and the spindle pole body. Curr Biol.

[b57] Fankhauser C, Marks J, Reymond A, Simanis V (1993). The *S. pombe cdc16* gene is required both for maintenance of p34cdc2 kinase activity and regulation of septum formation: a link between mitosis and cytokinesis?. EMBO J.

[b58] Fankhauser C, Reymond A, Cerutti L, Utzig S, Hofmann K, Simanis V (1995). The *S. pombe cdc15* gene is a key element in the reorganization of F-actin at mitosis. Cell.

[b59] Fankhauser C, Simanis V (1993). The *Schizosaccharomyces pombe cdc14* gene is required for septum formation and can also inhibit nuclear division. Mol Biol Cell.

[b60] Fankhauser C, Simanis V (1994). The cdc7 protein kinase is a dosage dependent regulator of septum formation in fission yeast. EMBO J.

[b61] Field CM, Alberts BM (1995). Anillin, a contractile ring protein that cycles from the nucleus to the cell cortex. J Cell Biol.

[b62] Fishkind DJ, Wang YL (1993). Orientation and three-dimensional organization of actin filaments in dividing cultured cells. J Cell Biol.

[b63] Foe VE, von Dassow G (2008). Stable and dynamic microtubules coordinately shape the myosin activation zone during cytokinetic furrow formation. J Cell Biol.

[b64] Fujiwara T, Bandi M, Nitta M, Ivanova EV, Bronson RT, Pellman D (2005). Cytokinesis failure generating tetraploids promotes tumorigenesis in p53-null cells. Nature.

[b65] Furge KA, Wong K, Armstrong J, Balasubramanian M, Albright CF (1998). Byr4 and Cdc16 form a two-component GTPase-activating protein for the Spg1 GTPase that controls septation in fission yeast. Curr Biol.

[b66] Furge KA, Cheng QC, Jwa M, Shin S, Song K, Albright CF (1999). Regions of Byr4, a regulator of septation in fission yeast, that bind Spg1 or Cdc16 and form a two-component GTPase-activating protein with Cdc16. J Biol Chem.

[b67] Gachet Y, Hyams JS (2005). Endocytosis in fission yeast is spatially associated with the actin cytoskeleton during polarised cell growth and cytokinesis. J Cell Sci.

[b68] Ganem NJ, Storchova Z, Pellman D (2007). Tetraploidy, aneuploidy and cancer. Curr Opin Genet Dev.

[b69] Garcia-Cortes JC, McCollum D (2009). Proper timing of cytokinesis is regulated by *Schizosaccharomyces pombe* Etd1. J Cell Biol.

[b70] Ge W, Chew TG, Wachtler V, Naqvi SN, Balasubramanian MK (2005). The novel fission yeast protein Pal1p interacts with Hip1-related Sla2p/End4p and is involved in cellular morphogenesis. Mol Biol Cell.

[b71] Gould KL, Simanis V (1997). The control of septum formation in fission yeast. Genes Dev.

[b72] Grallert A, Krapp A, Bagley S, Simanis V, Hagan IM (2004). Recruitment of NIMA kinase shows that maturation of the S. pombe spindle-pole body occurs over consecutive cell cycles and reveals a role for NIMA in modulating SIN activity. Genes Dev.

[b73] Gromley A, Jurczyk A, Sillibourne J, Halilovic E, Mogensen M, Groisman I, Blomberg M, Doxsey S (2003). A novel human protein of the maternal centriole is required for the final stages of cytokinesis and entry into S phase. J Cell Biol.

[b74] Gromley A, Yeaman C, Rosa J, Redick S, Chen CT, Mirabelle S, Guha M, Sillibourne J, Doxsey SJ (2005). Centriolin anchoring of exocyst and SNARE complexes at the midbody is required for secretory-vesicle-mediated abscission. Cell.

[b75] Guertin DA, McCollum D (2001). Interaction between the noncatalytic region of Sid1p kinase and Cdc14p is required for full catalytic activity and localization of Sid1p. J Biol Chem.

[b76] Guertin DA, Chang L, Irshad F, Gould KL, McCollum D (2000). The role of the sid1p kinase and cdc14p in regulating the onset of cytokinesis in fission yeast. EMBO J.

[b77] Guertin DA, Venkatram S, Gould KL, McCollum D (2002). Dma1 prevents mitotic exit and cytokinesis by inhibiting the septation initiation network (SIN). Dev Cell.

[b78] Guha M, Zhou M, Wang YL (2005). Cortical actin turnover during cytokinesis requires myosin II. Curr Biol.

[b79] Gunsalus KC, Bonaccorsi S, Williams E, Verni F, Gatti M, Goldberg ML (1995). Mutations in *twinstar*, a *Drosophila* gene encoding a cofilin/ADF homologue, result in defects in centrosome migration and cytokinesis. J Cell Biol.

[b80] Hachet O, Simanis V (2008). Mid1p/anillin and the septation initiation network orchestrate contractile ring assembly for cytokinesis. Genes Dev.

[b81] Hagan IM (1998). The fission yeast microtubule cytoskeleton. J Cell Sci.

[b82] Hagan IM, Hyams JS (1988). The use of cell division cycle mutants to investigate the control of microtubule distribution in the fission yeast *Schizosaccharomyces pombe*. J Cell Sci.

[b83] Harvey K, Tapon N (2007). The Salvador-Warts-Hippo pathway—an emerging tumour-suppressor network. Nat Rev Cancer.

[b84] He X, Patterson TE, Sazer S (1997). The *Schizosaccharomyces pombe* spindle checkpoint protein mad2p blocks anaphase and genetically interacts with the anaphase-promoting complex. Proc Natl Acad Sci USA.

[b85] He X, Jones MH, Winey M, Sazer S (1998). Mph1, a member of the Mps1-like family of dual specificity protein kinases, is required for the spindle checkpoint in *S. pombe*. J Cell Sci.

[b86] Heitz MJ, Petersen J, Valovin S, Hagan IM (2001). MTOC formation during mitotic exit in fission yeast. J Cell Sci.

[b87] Hergovich A, Hemmings BA (2009). Mammalian NDR/LATS protein kinases in hippo tumor suppressor signaling. Biofactors.

[b88] Hiramoto Y (1975). Force exerted by the cleavage furrow of Sea Urchin Eggs. Dev Growth Differ.

[b89] Hotulainen P, Lappalainen P (2006). Stress fibers are generated by two distinct actin assembly mechanisms in motile cells. J Cell Biol.

[b90] Hou MC, Salek J, McCollum D (2000). Mob1p interacts with the Sid2p kinase and is required for cytokinesis in fission yeast. Curr Biol.

[b91] Huang Y, Chew TG, Ge W, Balasubramanian MK (2007). Polarity determinants Tea1p, Tea4p, and Pom1p inhibit division-septum assembly at cell ends in fission yeast. Dev Cell.

[b92] Huang Y, Yan H, Balasubramanian MK (2008). Assembly of normal actomyosin rings in the absence of Mid1p and cortical nodes in fission yeast. J Cell Biol.

[b93] Humbel BM, Konomi M, Takagi T, Kamasawa N, Ishijima SA, Osumi M (2001). In situ localization of beta-glucans in the cell wall of *Schizosaccharomyces pombe*. Yeast.

[b94] Ishiguro J (1998). Genetic control of fission yeast cell wall synthesis: the genes involved in wall biogenesis and their interactions in *Schizosaccharomyces pombe*. Genes Genet Syst.

[b95] Jiang W, Hallberg RL (2001). Correct regulation of the septation initiation network in *Schizosaccharomyces pombe* requires the activities of *par1* and *par2*. Genetics.

[b96] Jin QW, McCollum D (2003). Scw1p antagonizes the septation initiation network to regulate septum formation and cell separation in the fission yeast *Schizosaccharomyces pombe*. Eukaryot Cell.

[b97] Jin QW, Zhou M, Bimbo A, Balasubramanian MK, McCollum D (2006). A role for the septation initiation network in septum assembly revealed by genetic analysis of *sid2-250* suppressors. Genetics.

[b98] Jin QW, Ray S, Choi SH, McCollum D (2007). The nucleolar Net1/Cfi1-related protein Dnt1 antagonizes the septation initiation network in fission yeast. Mol Biol Cell.

[b99] Jochová J, Rupes I, Streiblová E (1991). F-actin contractile rings in protoplasts of the yeast *Schizosaccharomyces*. Cell Biol Int Rep.

[b100] Jurgens G (2005). Plant cytokinesis: fission by fusion. Trends Cell Biol.

[b101] Kachur TM, Pilgrim DB (2008). Myosin assembly, maintenance and degradation in muscle: role of the chaperone UNC-45 in myosin thick filament dynamics. Int J Mol Sci.

[b102] Kachur T, Ao W, Berger J, Pilgrim D (2004). Maternal UNC-45 is involved in cytokinesis and colocalizes with non-muscle myosin in the early *Caenorhabditis elegans* embryo. J Cell Sci.

[b103] Kaji N, Ohashi K, Shuin M, Niwa R, Uemura T, Mizuno K (2003). Cell cycle-associated changes in Slingshot phosphatase activity and roles in cytokinesis in animal cells. J Biol Chem.

[b104] Kamasaki T, Osumi M, Mabuchi I (2007). Three-dimensional arrangement of F-actin in the contractile ring of fission yeast. J Cell Biol.

[b105] Kaminer B, Bell AL (1966). Myosin filamentogenesis: effects of pH and ionic concentration. J Mol Biol.

[b106] Kanada M, Nagasaki A, Uyeda TQ (2005). Adhesion-dependent and contractile ring-independent equatorial furrowing during cytokinesis in mammalian cells. Mol Biol Cell.

[b107] Kanai M, Kume K, Miyahara K, Sakai K, Nakamura K, Leonhard K, Wiley DJ, Verde F, Toda T, Hirata D (2005). Fission yeast MO25 protein is localized at SPB and septum and is essential for cell morphogenesis. EMBO J.

[b108] Kanbe T, Kobayashi I, Tanaka K (1989). Dynamics of cytoplasmic organelles in the cell cycle of the fission yeast *Schizosaccharomyces pombe*: three-dimensional reconstruction from serial sections. J Cell Sci.

[b109] Karagiannis J, Oulton R, Young PG (2002). The Scw1 RNA-binding domain protein regulates septation and cell-wall structure in fission yeast. Genetics.

[b110] Kitayama C, Sugimoto A, Yamamoto M (1997). Type II myosin heavy chain encoded by the *myo2* gene composes the contractile ring during cytokinesis in *Schizosaccharomyces pombe*. J Cell Biol.

[b111] Kovar DR, Kuhn JR, Tichy AL, Pollard TD (2003). The fission yeast cytokinesis formin Cdc12p is a barbed end actin filament capping protein gated by profilin. J Cell Biol.

[b112] Krapp A, Simanis V (2008). An overview of the fission yeast septation initiation network (SIN). Biochem Soc Trans.

[b113] Krapp A, Schmidt S, Cano E, Simanis V (2001). *S. pombe* cdc11p, together with sid4p, provides an anchor for septation initiation network proteins on the spindle pole body. Curr Biol.

[b114] Krapp A, Collin P, Cano del Rosario E, Simanis V (2008). Homoeostasis between the GTPase Spg1p and its GAP in the regulation of cytokinesis in *S. pombe*. J Cell Sci.

[b115] Lattmann E, Krapp A, Simanis V (2009). Cytokinesis: closure resets your SIN. Curr Biol.

[b116] Le Goff X, Woollard A, Simanis V (1999). Analysis of the *cps1* gene provides evidence for a septation checkpoint in *Schizosaccharomyces pombe*. Mol Gen Genet.

[b117] Le Goff X, Buvelot S, Salimova E, Guerry F, Schmidt S, Cueille N, Cano E, Simanis V (2001). The protein phosphatase 2A B′-regulatory subunit par1p is implicated in regulation of the *S. pombe* septation initiation network. FEBS Lett.

[b118] Lee MG, Nurse P (1987). Complementation used to clone a human homologue of the fission yeast cell cycle control gene *cdc2*. Nature.

[b119] Li C, Furge KA, Cheng QC, Albright CF (2000). Byr4 localizes to spindle-pole bodies in a cell cycle-regulated manner to control Cdc7 localization and septation in fission yeast. J Biol Chem.

[b120] Li R (2007). Cytokinesis in development and disease: variations on a common theme. Cell Mol Life Sci.

[b121] Lindas AC, Karlsson EA, Lindgren MT, Ettema TJ, Bernander R (2008). A unique cell division machinery in the *Archaea*. Proc Natl Acad Sci USA.

[b122] Liu J, Wang H, Balasubramanian MK (2000). A checkpoint that monitors cytokinesis in *Schizosaccharomyces pombe*. J Cell Sci.

[b123] Liu J, Tang X, Wang H, Oliferenko S, Balasubramanian MK (2002). The localization of the integral membrane protein Cps1p to the cell division site is dependent on the actomyosin ring and the septation-inducing network in *Schizosaccharomyces pombe*. Mol Biol Cell.

[b124] Lord M, Pollard TD (2004). UCS protein Rng3p activates actin filament gliding by fission yeast myosin-II. J Cell Biol.

[b125] Lord M, Laves E, Pollard TD (2005). Cytokinesis depends on the motor domains of myosin-II in fission yeast but not in budding yeast. Mol Biol Cell.

[b126] Lord M, Sladewski TE, Pollard TD (2008). Yeast UCS proteins promote actomyosin interactions and limit myosin turnover in cells. Proc Natl Acad Sci USA.

[b127] Mabuchi I, Itoh T, Sugi H (1992).

[b128] Magidson V, Chang F, Khodjakov A (2006). Regulation of cytokinesis by spindle-pole bodies. Nat Cell Biol.

[b129] Marks J, Hagan IM, Hyams JS (1986). Growth polarity and cytokinesis in fission yeast: the role of the cytoskeleton. J Cell Sci Suppl.

[b130] Marks J, Fankhauser C, Simanis V (1992). Genetic interactions in the control of septation in *Schizosaccharomyces pombe*. J Cell Sci.

[b131] Martin-Cuadrado AB, Morrell JL, Konomi M, An H, Petit C, Osumi M, Balasubramanian M, Gould KL, Del Rey F, de Aldana CR (2005). Role of septins and the exocyst complex in the function of hydrolytic enzymes responsible for fission yeast cell separation. Mol Biol Cell.

[b132] Martin-Garcia R, Valdivieso MH (2006). The fission yeast Chs2 protein interacts with the type-II myosin Myo3p and is required for the integrity of the actomyosin ring. J Cell Sci.

[b133] Martin SG (2009). Geometric control of the cell cycle. Cell Cycle.

[b134] Martin SG, Berthelot-Grosjean M (2009). Polar gradients of the DYRK-family kinase Pom1 couple cell length with the cell cycle. Nature.

[b135] Matallanas D, Romano D, Hamilton G, Kolch W, O'Neill E (2008). A Hippo in the ointment: MST signalling beyond the fly. Cell Cycle.

[b136] Matsuyama A, Arai R, Yashiroda Y, Shirai A, Kamata A, Sekido S, Kobayashi Y, Hashimoto A, Hamamoto M, Hiraoka Y (2006). ORFeome cloning and global analysis of protein localization in the fission yeast *Schizosaccharomyces pombe*. Nat Biotechnol.

[b137] Maupin P, Pollard TD (1986). Arrangement of actin filaments and myosin-like filaments in the contractile ring and of actin-like filaments in the mitotic spindle of dividing HeLa cells. J Ultrastruct Mol Struct Res.

[b138] Mayer C, Filopei J, Batac J, Alford L, Paluh JL (2006). An extended anaphase signaling pathway for Mad2p includes microtubule organizing center proteins and multiple motor-dependent transitions. Cell Cycle.

[b139] McCollum D, Feoktistova A, Morphew M, Balasubramanian M, Gould KL (1996). The *Schizosaccharomyces pombe* actin-related protein, Arp3, is a component of the cortical actin cytoskeleton and interacts with profilin. EMBO J.

[b140] McCully EK, Robinow CF (1971). Mitosis in the fission yeast *Schizosaccharomyces pombe*: a comparative study with light and electron microscopy. J Cell Sci.

[b141] Mehta S, Gould KL (2006). Identification of functional domains within the septation initiation network kinase, Cdc7. J Biol Chem.

[b142] Mendoza M, Redemann S, Brunner D (2005). The fission yeast MO25 protein functions in polar growth and cell separation. Eur J Cell Biol.

[b143] Minet M, Nurse P, Thuriaux P, Mitchison JM (1979). Uncontrolled septation in a cell division cycle mutant of the fission yeast *Schizosaccharomyces pombe*. J Bacteriol.

[b144] Mishima M, Kaitna S, Glotzer M (2002). Central spindle assembly and cytokinesis require a kinesin-like protein/RhoGAP complex with microtubule bundling activity. Dev Cell.

[b145] Mishima M, Pavicic V, Gruneberg U, Nigg EA, Glotzer M (2004). Cell cycle regulation of central spindle assembly. Nature.

[b146] Mishra M, Oliferenko S (2008). Cytokinesis: catch and drag. Curr Biol.

[b147] Mishra M, Karagiannis J, Trautmann S, Wang H, McCollum D, Balasubramanian MK (2004). The Clp1p/Flp1p phosphatase ensures completion of cytokinesis in response to minor perturbation of the cell division machinery in *Schizosaccharomyces pombe*. J Cell Sci.

[b148] Mitchison JM, Nurse P (1985). Growth in cell length in the fission yeast *Schizosaccharomyces pombe*. J Cell Sci.

[b149] Morrell JL, Tomlin GC, Rajagopalan S, Venkatram S, Feoktistova AS, Tasto JJ, Mehta S, Jennings JL, Link A, Balasubramanian MK (2004). Sid4p-Cdc11p assembles the septation initiation network and its regulators at the *S. pombe* SPB. Curr Biol.

[b150] Moseley JB, Mayeux A, Paoletti A, Nurse P (2009). A spatial gradient coordinates cell size and mitotic entry in fission yeast. Nature.

[b151] Motegi F, Nakano K, Mabuchi I (2000). Molecular mechanism of myosin-II assembly at the division site in *Schizosaccharomyces pombe*. J Cell Sci.

[b152] Motegi F, Arai R, Mabuchi I (2002). Identification of two type V myosins in fission yeast, one of which functions in polarized cell growth and moves rapidly in the cell. Mol Cell Biol.

[b153] Motegi F, Mishra M, Balasubramanian MK, Mabuchi I (2004). Myosin-II reorganization during mitosis is controlled temporally by its dephosphorylation and spatially by Mid1 in fission yeast. J Cell Biol.

[b154] Mukhina S, Wang YL, Murata-Hori M (2007). Alpha-actinin is required for tightly regulated remodeling of the actin cortical network during cytokinesis. Dev Cell.

[b155] Mulvihill DP, Petersen J, Ohkura H, Glover DM, Hagan IM (1999). Plo1 kinase recruitment to the spindle pole body and its role in cell division in *Schizosaccharomyces pombe*. Mol Biol Cell.

[b156] Mulvihill DP, Barretto C, Hyams JS (2001). Localization of fission yeast type II myosin, Myo2, to the cytokinetic actin ring is regulated by phosphorylation of a C-terminal coiled-coil domain and requires a functional septation initiation network. Mol Biol Cell.

[b157] Mulvihill DP, Edwards SR, Hyams JS (2006). A critical role for type V myosin, Myo52, in septum deposition and cell fission during cytokinesis in *Schizosaccharomyces pombe*. Cell Motility Cytoskel.

[b158] Murone M, Simanis V (1996). The fission yeast *dma1* gene is a component of the spindle assembly checkpoint, required to prevent septum formation and premature exit from mitosis if spindle function is compromised. EMBO J.

[b159] Murthy K, Wadsworth P (2005). Myosin-II-dependent localization and dynamics of F-actin during cytokinesis. Curr Biol.

[b160] Nabeshima K, Nakagawa T, Straight AF, Murray A, Chikashige Y, Yamashita YM, Hiraoka Y, Yanagida M (1998). Dynamics of centromeres during metaphase-anaphase transition in fission yeast: Dis1 is implicated in force balance in metaphase bipolar spindle. Mol Biol Cell.

[b161] Nakano K, Mabuchi I (2006). Actin-depolymerizing protein Adf1 is required for formation and maintenance of the contractile ring during cytokinesis in fission yeast. Mol Biol Cell.

[b162] Nakano K, Satoh K, Morimatsu A, Ohnuma M, Mabuchi I (2001). Interactions among a fimbrin, a capping protein, and an actin-depolymerizing factor in organization of the fission yeast actin cytoskeleton. Mol Biol Cell.

[b163] Nakano K, Imai J, Arai R, Toh EA, Matsui Y, Mabuchi I (2002). The small GTPase Rho3 and the diaphanous/formin For3 function in polarized cell growth in fission yeast. J Cell Sci.

[b164] Nakano K, Bunai F, Numata O (2005). Stg 1 is a novel SM22/transgelin-like actin-modulating protein in fission yeast. FEBS Lett.

[b165] Naqvi NI, Eng K, Gould KL, Balasubramanian MK (1999). Evidence for F-actin-dependent and -independent mechanisms involved in assembly and stability of the medial actomyosin ring in fission yeast. EMBO J.

[b166] Neujahr R, Heizer C, Gerisch G (1997). Myosin II-independent processes in mitotic cells of *Dictyostelium discoideum*: redistribution of the nuclei, re-arrangement of the actin system and formation of the cleavage furrow. J Cell Sci.

[b167] Nishimura Y, Mabuchi I (2003). An IQGAP-like protein is involved in actin assembly together with Cdc42 in the sea urchin egg. Cell Motil Cytoskeleton.

[b168] Noguchi T, Arai R, Motegi F, Nakano K, Mabuchi I (2001). Contractile ring formation in *Xenopus* egg and fission yeast. Cell Struct Funct.

[b169] O'Connell CB, Warner AK, Wang Y (2001). Distinct roles of the equatorial and polar cortices in the cleavage of adherent cells. Curr Biol.

[b170] Oegema K, Savoian MS, Mitchison TJ, Field CM (2000). Functional analysis of a human homologue of the *Drosophila* actin binding protein anillin suggests a role in cytokinesis. J Cell Biol.

[b171] Ohkura H, Hagan IM, Glover DM (1995). The conserved *Schizosaccharomyces pombe* kinase *plo1*, required to form a bipolar spindle, the actin ring, and septum, can drive septum formation in G1 and G2 cells. Genes Dev.

[b172] Oliferenko S, Chew TG, Balasubramanian MK (2009). Positioning cytokinesis. Genes Dev.

[b173] Ono K, Parast M, Alberico C, Benian GM, Ono S (2003). Specific requirement for two ADF/cofilin isoforms in distinct actin-dependent processes in *Caenorhabditis elegans*. J Cell Sci.

[b174] Otegui MS, Verbrugghe KJ, Skop AR (2005). Midbodies and phragmoplasts: analogous structures involved in cytokinesis. Trends Cell Biol.

[b175] Padte NN, Martin SG, Howard M, Chang F (2006). The cell-end factor pom1p inhibits mid1p in specification of the cell division plane in fission yeast. Curr Biol.

[b176] Pantaloni D, Le Clainche C, Carlier MF (2001). Mechanism of actin-based motility. Science.

[b177] Paoletti A, Chang F (2000). Analysis of mid1p, a protein required for placement of the cell division site, reveals a link between the nucleus and the cell surface in fission yeast. Mol Biol Cell.

[b178] Pardo M, Nurse P (2003). Equatorial retention of the contractile actin ring by microtubules during cytokinesis. Science.

[b179] Pelham RJ, Chang F (2002). Actin dynamics in the contractile ring during cytokinesis in fission yeast. Nature.

[b180] Petersen J, Hagan IM (2005). Polo kinase links the stress pathway to cell cycle control and tip growth in fission yeast. Nature.

[b181] Petersen J, Nurse P (2007). TOR signalling regulates mitotic commitment through the stress MAP kinase pathway and the Polo and Cdc2 kinases. Nat Cell Biol.

[b182] Petit CS, Mehta S, Roberts RH, Gould KL (2005). Ace2p contributes to fission yeast septin ring assembly by regulating **mid2**+ expression. J Cell Sci.

[b183] Petronczki M, Lenart P, Peters JM (2008). Polo on the rise-from mitotic entry to cytokinesis with Plk1. Dev Cell.

[b184] Pinar M, Coll PM, Rincon SA, Perez P (2008). *Schizosaccharomyces pombe* Pxl1 is a paxillin homologue that modulates Rho1 activity and participates in cytokinesis. Mol Biol Cell.

[b185] Pollard TD (2008). Progress towards understanding the mechanism of cytokinesis in fission yeast. Biochem Soc Trans.

[b186] Pollard TD (2010). Mechanics of cytokinesis in eukaryotes. Curr Opin Cell Biol.

[b187] Pollard TD, Wu JQ (2010). Understanding cytokinesis: lessons from fission yeast. Nat Rev Mol Cell Biol.

[b188] Prekeris R, Gould GW (2008). Breaking up is hard to do - membrane traffic in cytokinesis. J Cell Sci.

[b189] Rappaport R (1967). Cell division: Direct measurement of maximum tension exerted by furrow of *Echinoderm* eggs. Science.

[b190] Ray S, Kume K, Gupta S, Ge W, Balasubramanian M, Hirata D, McCollum D (2010). The mitosis-to-interphase transition is coordinated by cross talk between the SIN and MOR pathways in *Schizosaccharomyces pombe*. J Cell Biol.

[b191] Roberts-Galbraith RH, Gould KL (2008). Stepping into the ring: the SIN takes on contractile ring assembly. Genes Dev.

[b192] Roberts-Galbraith RH, Ohi MD, Ballif BA, Chen JS, McLeod I, McDonald WH, Gygi SP, Yates JR, Gould KL (2010). Dephosphorylation of F-BAR protein Cdc15 modulates its conformation and stimulates its scaffolding activity at the cell division site. Mol Cell.

[b193] Rock JM, Amon A (2009). The FEAR network. Curr Biol.

[b194] Rosenberg JA, Tomlin GC, McDonald WH, Snydsman BE, Muller EG, Yates JR, Gould KL (2006). Ppc89 links multiple proteins, including the septation initiation network, to the core of the fission yeast spindle-pole body. Mol Biol Cell.

[b195] Salimova E, Sohrmann M, Fournier N, Simanis V (2000). The *S. pombe* orthologue of the *S. cerevisiae mob1* gene is essential and functions in signalling the onset of septum formation. J Cell Sci.

[b196] Sanger JM, Sanger JW (1980). Banding and polarity of actin filaments in interphase and cleaving cells. J Cell Biol.

[b197] Satterwhite LL, Pollard TD (1992). Cytokinesis. Curr Opin Cell Biol.

[b198] Schmidt S, Sohrmann M, Hofmann K, Woollard A, Simanis V (1997). The Spg1p GTPase is an essential, dosage-dependent inducer of septum formation in *Schizosaccharomyces pombe*. Genes Dev.

[b199] Schroeder TE (1972). The contractile ring. II. Determining its brief existence, volumetric changes, and vital role in cleaving *Arbacia* eggs. J Cell Biol.

[b200] Severson AF, Baillie DL, Bowerman B (2002). A Formin Homology protein and a profilin are required for cytokinesis and Arp2/3-independent assembly of cortical microfilaments in *C. elegans*. Curr Biol.

[b201] Simanis V (2003). Events at the end of mitosis in the budding and fission yeasts. J Cell Sci.

[b202] Sipiczki M (2007). Splitting of the fission yeast septum. FEMS Yeast Res.

[b203] Sladewski TE, Previs MJ, Lord M (2009). Regulation of fission yeast myosin-II function and contractile ring dynamics by regulatory light-chain and heavy-chain phosphorylation. Mol Biol Cell.

[b204] Sohrmann M, Fankhauser C, Brodbeck C, Simanis V (1996). The *dmf1*/*mid1* gene is essential for correct positioning of the division septum in fission yeast. Genes Dev.

[b205] Sohrmann M, Schmidt S, Hagan I, Simanis V (1998). Asymmetric segregation on spindle poles of the *Schizosaccharomyces pombe* septum-inducing protein kinase Cdc7p. Genes Dev.

[b206] Somma MP, Fasulo B, Cenci G, Cundari E, Gatti M (2002). Molecular dissection of cytokinesis by RNA interference in *Drosophila* cultured cells. Mol Biol Cell.

[b207] Song K, Mach KE, Chen CY, Reynolds T, Albright CF (1996). A novel suppressor of *ras1* in fission yeast, *byr4*, is a dosage- dependent inhibitor of cytokinesis. J Cell Biol.

[b208] Sparks CA, Morphew M, McCollum D (1999). Sid2p, a spindle pole body kinase that regulates the onset of cytokinesis. J Cell Biol.

[b209] Stark BC, Sladewski TE, Pollard LW, Lord M (2010). Tropomyosin and myosin-II cellular levels promote actomyosin ring assembly in fission yeast. Mol Biol Cell.

[b210] Stegmeier F, Amon A (2004). Closing mitosis: the functions of the Cdc14 phosphatase and its regulation. Annu Rev Genet.

[b211] Straight AF, Cheung A, Limouze J, Chen I, Westwood NJ, Sellers JR, Mitchison TJ (2003). Dissecting temporal and spatial control of cytokinesis with a myosin II Inhibitor. Science.

[b212] Straight AF, Field CM, Mitchison TJ (2005). Anillin binds nonmuscle myosin II and regulates the contractile ring. Mol Biol Cell.

[b213] Streiblova E, Hasek J, Jelke E (1984). Septum pattern in ts mutants of *Schizosaccharomyces pombe* defective in genes *cdc3, cdc4, cdc8* and *cdc12*. J Cell Sci.

[b214] Takaine M, Numata O, Nakano K (2009). Fission yeast IQGAP arranges actin filaments into the cytokinetic contractile ring. EMBO J.

[b215] Tanaka K, Petersen J, MacIver F, Mulvihill DP, Glover DM, Hagan IM (2001). The role of Plo1 kinase in mitotic commitment and septation in *Schizosaccharomyces pombe*. EMBO J.

[b216] Tasto JJ, Morrell JL, Gould KL (2003). An anillin homologue, Mid2p, acts during fission yeast cytokinesis to organize the septin ring and promote cell separation. J Cell Biol.

[b217] Tatebe H, Nakano K, Maximo R, Shiozaki K (2008). Pom1 DYRK regulates localization of the Rga4 GAP to ensure bipolar activation of Cdc42 in fission yeast. Curr Biol.

[b218] Tomlin GC, Morrell JL, Gould KL (2002). The spindle pole body protein cdc11p links sid4p to the fission yeast septation initiation network. Mol Biol Cell.

[b219] Tran PT, Doye V, Chang F, Inoue S (2000). Microtubule-dependent nuclear positioning and nuclear-dependent septum positioning in the fission yeast *Schizosaccharomyces* [correction of *Saccharomyces*] *pombe*. Biol Bull.

[b220] Trautmann S, Wolfe BA, Jorgensen P, Tyers M, Gould KL, McCollum D (2001). Fission yeast Clp1p phosphatase regulates G2/M transition and coordination of cytokinesis with cell cycle progression. Curr Biol.

[b221] Vale RD, Spudich JA, Griffis ER (2009). Dynamics of myosin, microtubules, and Kinesin-6 at the cortex during cytokinesis in *Drosophila* S2 cells. J Cell Biol.

[b222] Vardy L, Fujita A, Toda T (2002). The gamma-tubulin complex protein Alp4 provides a link between the metaphase checkpoint and cytokinesis in fission yeast. Genes Cells.

[b223] Vavylonis D, Wu JQ, Hao S, O'Shaughnessy B, Pollard TD (2008). Assembly mechanism of the contractile ring for cytokinesis by fission yeast. Science.

[b224] Verkhovsky AB, Svitkina TM, Borisy GG (1995). Myosin II filament assemblies in the active lamella of fibroblasts: their morphogenesis and role in the formation of actin filament bundles. J Cell Biol.

[b225] Wachtler V, Huang Y, Karagiannis J, Balasubramanian MK (2006). Cell cycle-dependent roles for the FCH-domain protein Cdc15p in formation of the actomyosin ring in *Schizosaccharomyces pombe*. Mol Biol Cell.

[b226] Wang H, Tang X, Liu J, Trautmann S, Balasundaram D, McCollum D, Balasubramanian MK (2002). The multiprotein exocyst complex is essential for cell separation in *Schizosaccharomyces pombe*. Mol Cell Biol.

[b227] Wang YL (2005). The mechanism of cortical ingression during early cytokinesis: thinking beyond the contractile ring hypothesis. Trends Cell Biol.

[b228] Watanabe S, Ando Y, Yasuda S, Hosoya H, Watanabe N, Ishizaki T, Narumiya S (2008). mDia2 induces the actin scaffold for the contractile ring and stabilizes its position during cytokinesis in NIH 3T3 cells. Mol Biol Cell.

[b229] Werner M, Glotzer M (2008). Control of cortical contractility during cytokinesis. Biochem Soc Trans.

[b230] Wolfe BA, Gould KL (2005). Split decisions: coordinating cytokinesis in yeast. Trends Cell Biol.

[b231] Wolfe BA, McDonald WH, Yates JR, Gould KL (2006). Phospho-regulation of the Cdc14/Clp1 phosphatase delays late mitotic events in *S. pombe*. Dev Cell.

[b232] Wolfe BA, Takaki T, Petronczki M, Glotzer M (2009). Polo-like kinase 1 directs assembly of the HsCyk-4 RhoGAP/Ect2 RhoGEF complex to initiate cleavage furrow formation. PLoS Biol.

[b233] Wong KC, D'Souza VM, Naqvi NI, Motegi F, Mabuchi I, Balasubramanian MK (2002). Importance of a myosin II-containing progenitor for actomyosin ring assembly in fission yeast. Curr Biol.

[b234] Wong KC, Naqvi NI, Iino Y, Yamamoto M, Balasubramanian MK (2000). Fission yeast Rng3p: an UCS-domain protein that mediates myosin II assembly during cytokinesis. J Cell Sci.

[b235] Wu JQ, Pollard TD (2005). Counting cytokinesis proteins globally and locally in fission yeast. Science.

[b236] Wu JQ, Kuhn JR, Kovar DR, Pollard TD (2003). Spatial and temporal pathway for assembly and constriction of the contractile ring in fission yeast cytokinesis. Dev Cell.

[b237] Wu JQ, Sirotkin V, Kovar DR, Lord M, Beltzner CC, Kuhn JR, Pollard TD (2006). Assembly of the cytokinetic contractile ring from a broad band of nodes in fission yeast. J Cell Biol.

[b238] Yamano H, Gannon J, Hunt T (1996). The role of proteolysis in cell cycle progression in *Schizosaccharomyces pombe*. EMBO J.

[b239] Yasuda T, Numata O, Ohnishi K, Watanabe Y (1980). A contractile ring and cortical changes found in the dividing *Tetrahymena pyriformis*. Exp Cell Res.

[b240] Yonetani A, Chang F (2010). Regulation of cytokinesis by the formin cdc12p. Curr Biol.

[b241] Yonetani A, Lustig RJ, Moseley JB, Takeda T, Goode BL, Chang F (2008). Regulation and targeting of the fission yeast formin cdc12p in cytokinesis. Mol Biol Cell.

[b242] Yumura S, Fukui Y (1985). Reversible cyclic AMP-dependent change in distribution of myosin thick filaments in Dictyostelium. Nature.

[b243] Yumura S, Ueda M, Sako Y, Kitanishi-Yumura T, Yanagida T (2008). Multiple mechanisms for accumulation of myosin II filaments at the equator during cytokinesis. Traffic.

[b244] Zhang D, Vjestica A, Oliferenko S (2010). The cortical ER network limits the permissive zone for actomyosin ring assembly. Curr Biol.

[b245] Zhang L, Yue T, Jiang J (2009). Hippo signaling pathway and organ size control. Fly [Austin].

[b246] Zhou M, Wang YL (2008). Distinct pathways for the early recruitment of myosin II and actin to the cytokinetic furrow. Mol Biol Cell.

[b247] Zumdieck A, Kruse K, Bringmann H, Hyman AA, Julicher F (2007). Stress generation and filament turnover during actin ring constriction. PLoS One.

